# Assessment of diesel-contaminated domestic wastewater treated by constructed wetlands for irrigation of chillies grown in a greenhouse

**DOI:** 10.1007/s11356-016-7706-x

**Published:** 2016-09-27

**Authors:** Rawaa H. K. Al-Isawi, Miklas Scholz, Furat A. M. Al-Faraj

**Affiliations:** 1 Civil Engineering Research Group, School of Computing, Science and Engineering, The University of Salford, Newton Building, Salford, England M5 4WT UK; 2 Division of Water Resources Engineering, Faculty of Engineering, Lund University, P.O. Box 118, 221 00 Lund, Sweden

**Keywords:** Agricultural water resources management, *Capsicum annuum*, Ecological sanitation, Environmental pollution, Hydrocarbon contamination, Nutrient and trace mineral control, Reed bed, Water reclamation

## Abstract

In order to avoid environmental pollution and eliminate the need for using fertiliser, this study assessed for the first time the optimum performance of mature (in operation since 2011) vertical flow constructed wetlands in treating domestic wastewater (with and without hydrocarbon) and the subsequent recycling of the outflow to irrigate chillies (De Cayenne; *Capsicum annuum* (Linnaeus) Longum Group ‘De Cayenne’) grown in a greenhouse. Various variables were investigated to assess the treatment performance. Concerning chilli fruit numbers, findings showed that the highest fruit yields for all wetland filters were associated with those that received inflow wastewater with a high loading rate, reflecting the high nutrient availability in treated wastewater, which is of obvious importance for yield production. Findings also indicated that wetlands without hydrocarbon, small aggregate size, low contact time and low inflow loading rate provided high marketable yields (expressed in economic return). In comparison, chillies irrigated by filters with hydrocarbon contamination, small aggregate size, high contact time and high loading rate also resulted in high marketable yields of chillies, which pointed out the role of high contact time and high inflow load for better diesel degradation rates.

**Electronic supplementary material:**

The online version of this article (doi:10.1007/s11356-016-7706-x) contains supplementary material, which is available to authorized users.

## Introduction

Due to a rising demand in freshwater, water shortage-related problems have been growing in the world and freshwater resources are increasingly insufficient to satisfy a growing demand. Water scarcity and droughts have been increasingly becoming key concerns worldwide, not only in dry regions, but also in regions where freshwater resources are plenty (FAO 2012). Instead of potable water and natural freshwater, treated wastewater can be applied for agriculture, urban and industrial applications, recreational and ecosystem service needs and artificial recharge of belowground water (Asano et al. 2007; Al-Hamaiedeh and Bino 2010; Marinho et al. 2013). Recycling of treated urban wastewater for irrigation has been considered as one of the promising strategies in the agriculture sector (Aiello et al. 2007; Cirelli et al. 2012; Norton-Brandão et al. 2013).

Wetland treatment systems are primarily constructed to treat a range of wastewaters such as domestic wastewater, industrial effluents, urban and agricultural runoff, animal wastewaters and mine drainage (Scholz 2010, 2015; Sani et al. 2013a; Vymazal 2014; Al-Isawi et al. 2015a). For example, Dong et al. (2011) have underlined that some large-scale wetland schemes (e.g. integrated constructed wetlands operated in Glaslough, Ireland, between 2008 and 2010) have successfully treated domestic wastewater. Mean concentration removal efficiencies were high for chemical oxygen demand (COD), 5 days at 20 °C N-allylthiourea biochemical oxygen demand (BOD), total suspended solids (TSS), ammonia-nitrogen (NH_4_-N), nitrate-nitrogen (NO_3_-N), total nitrogen (TN) and molybdate reactive phosphate (MRP).

The benefits of constructed wetland systems for agricultural purposes are widely known. They produce water with adequate quality for irrigation (Cui et al. 2003; Morari and Giardini 2009; Becerra-Castro et al. 2015) and offer good removal rates concerning microbial contamination (Wu et al. 2016). This practice potentially increases agricultural yields, preserves freshwater, offsets the demand for chemical fertilisers and reduces the costs of wastewater treatment by avoiding nutrient removal units (Murray and Ray 2010). Contaminated wastewater with undesirable concentrations of hydrocarbons from oil spills linked to urban runoff or industrial discharge is a more recent challenge for agricultural application purposes (Scholz 2010; García-Delgado et al. 2012). However, studies showed low impact of treated domestic wastewater contaminated with hydrocarbon on soil and pepper fruit quality in a greenhouse environment (García-Delgado et al. 2012).

Indeed, constructed wetlands have successfully been adopted to treat wastewater contaminated with hydrocarbons (De Biase et al. 2011; Al-Baldawi et al. 2015; Al-Isawi et al. 2015a). De Biase et al. (2011) noted that volatilisation in vertical flow constructed wetlands is an important removal process for volatile hydrophobic compounds. Al-Baldawi et al. (2015) found that wetland plants contributed to the aeration of the rhizosphere, which in turn stimulates the activity of the rhizobacteria, subsequently leading to the high removal of diesel (>70 %) in subsurface flow constructed wetlands. Moreover, Al-Isawi et al. (2015a) showed that all petroleum hydrocarbon components treated in vertical flow wetland filters were highly degraded (>80 %).

Nickels (2012) pointed out that chillies need nearly 100 days to reach maturity and like warm, moist and nutrient-rich soil, as well as loamy soil that is neutral to weakly alkaline. The germination time is commonly between 5 days and 2 weeks. The sowing to cropping time is about 126 days. Chillies are perennial in subtropical and tropical regions, but they are usually grown as annuals in temperate climates (Nickels 2012).

The major minerals affecting the growth of plants are nitrogen, phosphorus, potassium, calcium, magnesium and sulphur. Aluminium, copper, iron, manganese, molybdenum and zinc are often described as heavy metals. For pH values of less than 7, heavy metals may be a challenge to sensitive plants according to FAO (2003).

FAO (1994) defined some ranges for the suitable use of treated domestic wastewater for recycling with regard to contaminants. Between 0 and 5 mg/l, between 0 and 2 mg/l and between 0 and 2 mg/l are considered acceptable ranges for the nutrients NH_4_-N, orthophosphate-phosphorous and potassium, respectively. Pescod (1992) pointed out that there is no restriction to reuse the treated wastewater in irrigation, if NO_3_-N values are <5 mg/l. Furthermore, FAO (2003) stated the nutrient requirements for pepper required for proper canopy formation and Haifa Chemicals (2014) published the required rates of macro and secondary plant nutrient uptake by pepper plants in greenhouses.

In order to mitigate environmental pollution, the sustainable treatment of urban wastewater with wetlands is a practical solution in most climatic zones. Moreover, the smart reuse of treated wastewater in agriculture eliminates the need for using fertiliser and protects soil from contamination by nutrients and trace metals. The application of pretreated wastewater in agriculture has not been efficiently managed, particularly in developing countries (Almuktar et al. 2015).

Almuktar et al. (2015) tried to recycle domestic wastewater treated by wetlands for irrigation of chillies and sweet peppers. However, this study received a lot of criticism, because it was undertaken in a laboratory under less than ideal and unrealistic boundary conditions, which have led to rather poor harvests. Moreover, the sample sizes were rather small. Therefore, this follow-up study avoided past shortcomings and is based on a statistically more representative set-up of greenhouse-grown chillies instead.

This article aims to assess for the first time the performance of diverse mature vertical flow constructed wetlands in treating domestic wastewater (with and without hydrocarbon contamination) for subsequent reuse in irrigation of chillies (De Cayenne; *Capsicum annuum* (Linnaeus) Longum Group ‘De Cayenne’), which are grown in a greenhouse. The key objectives are to examine the influence of differently treated wastewater (subject to the constructed wetland type) on the growth of chilli plants and the effect of temperature and relative humidity on the growth environment. Moreover, the amount of treated wastewater needed for irrigation and the economic viability of various experimental set-ups regarding their corresponding harvest (economic return and potential contamination by elements) and the impact of treated hydrocarbon water on chilli growth are also considered.

This study offers a promising solution to treat and subsequently reuse domestic wastewater in a more sustainable way, even when financial sources are limited. In addition, food grown on soil irrigated by pretreated wastewater offers an additional economic return, also helping to solve food shortages in many developed countries. Therefore, this study should be of interest to the international reader trying to protect the environment from pollution and solve wastewater treatment and food challenges at the same time.

## Materials and methodologies

### Set-up and operation of the constructed wetlands

The set-up of vertical flow constructed wetlands consists of ten filters (planted with *Phragmites australis* (Cav.) Trin. ex Steud. (Common Reed)), which were constructed from Pyrex tubes with an inner diameter of 19.5 cm and a height of 120 cm. The surface area of each wetland was approximately 300 cm^2^ to keep the experimental wetland operation manageable in terms of volumes to be treated. The relatively small size is justified considering that microbial treatment processes take place on a millimetre scale. Moreover, the use of small columns has been commonly reported in the peer-reviewed literature (Tang et al. 2009; Sani et al. 2013a, b; Al-Isawi et al. 2015a, b; Almuktar et al. 2015; Scholz 2010, 2015).

**Fig. 1:**
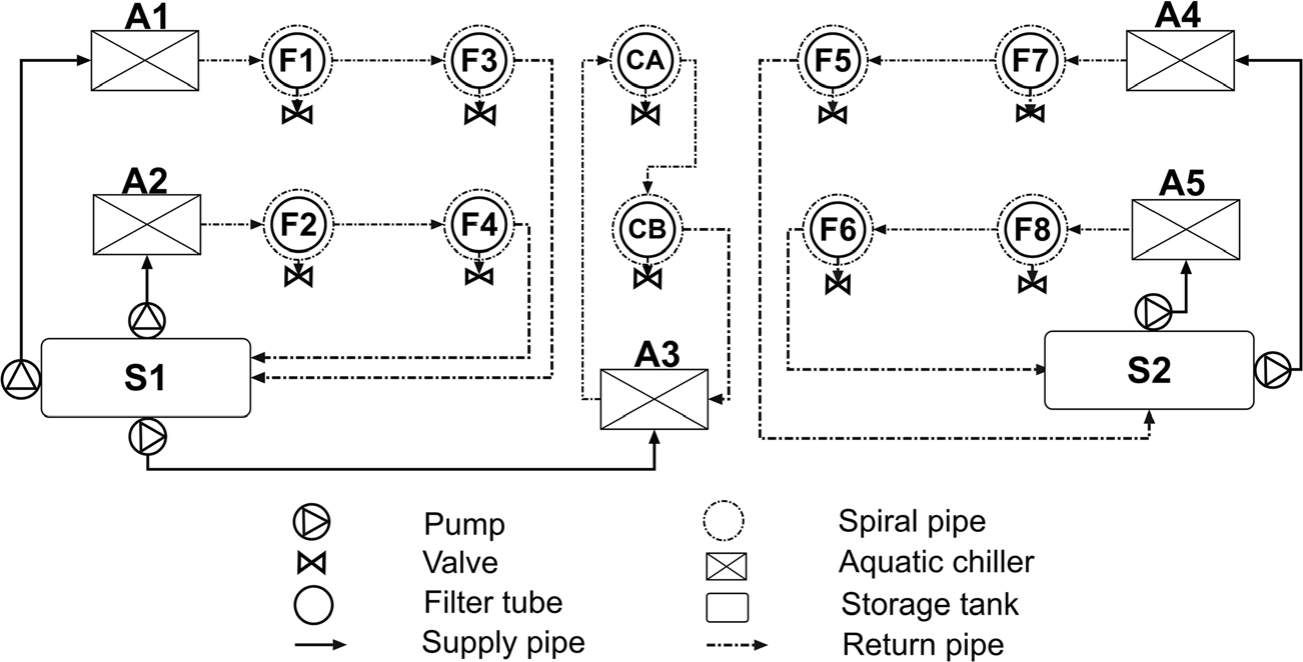
A schematic diagram (*top view*) of the experimental wetland system

**Fig. 2:**
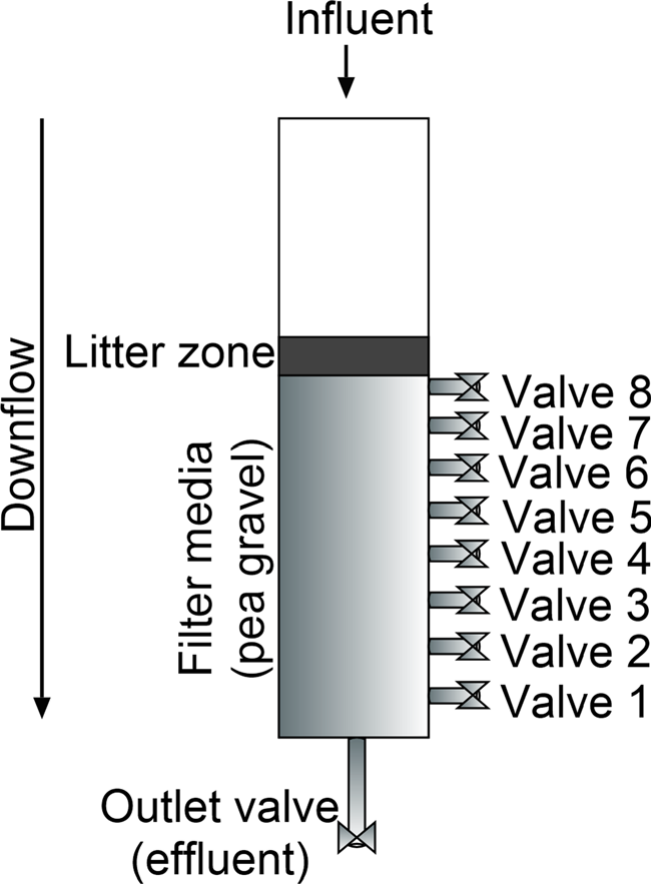
Side view of a wetland filter

All filters were filled with siliceous pea gravel up to a depth of 60 cm and were operated between 27 June 2011 and 24 December 2014 as described by Sani et al. (2013a) and are outlined in Fig. 1. The experimental set-up is indicated in Table 1, and Fig. 2 shows the side view of any wetland filter.

**Table 1:** Set-up of the experimental vertical flow wetland system

Filters	Aggregate diameter (mm)	Contact time (h)	Resting time (h)	COD^a^ load (mg/l)	COD^a^ loading rate	
					Mean (g/m^2^/day)	Standard deviation (g/m^2^/day)
Filter 1	20	72	48	137.9	6.3	2.8
Filter 2	20	72	48	137.9	6.3	2.8
Filter 3	10	72	48	137.9	6.3	2.8
Filter 4	10	72	48	137.9	6.3	2.8
Filter 5	10	72	48	280.3	12.7	5.6
Filter 6	10	72	48	280.3	12.7	5.6
Filter 7	10	36	48	137.9	8.1	3.6
Filter 8	10	36	24	137.9	10.6	4.8
Control A	10	72	48	2.3	0.1	0.1
Control B	10	72	48	2.3	0.1	0.1

After Al-Isawi et al. (2015a). The yearly average inflow volume to wetland system: filters 1 to 6 as well as controls A and B, 495 l/a; filter 7, 642 l/a filter 8, 839 l/a. On 26 September 2013 and 26 September 2014, 130 and 975 g of diesel, respectively, were added to filters 1, 3, 5 and control A

^a^Chemical oxygen demand

Four factors were investigated to examine the performance of the vertical flow constructed wetlands (Table 1): (1) gravel size, (2) contact time (also known as hydraulic detention time), (3) rest time and (4) inflow COD load. Filters 1 and 2 are compared to filters 3 and 4 to examine the impact of a larger aggregate diameter. Moreover, filters 5 and 6 are compared to filters 3 and 4 to estimate the influence of a higher inflow COD load. The selection of a lower contact time was investigated through comparing filter 7 with filters 3 and 4, and finally, the impact of rest time was obtained through a comparison between filter 7 and filter 8.

Wetland filters received 6.5 l of inflow water during feeding (Table 1). Filters 1 to 6 were tested after 72-h contact time and then left to rest for 48 h, while filters 7 and 8 were sampled after 36-h contact time and left to rest for 48 and 24 h in this order.

The preliminarily treated wastewater used for the inflow water was obtained from the local Davyhulme Sewage Works. Fresh wastewater was sampled regularly nearly once a week and was stored and aerated by aquarium air pumps in a dedicated cold room (Table 2). The quality of wastewater was considerably variable reflecting the variable nature of real domestic wastewater, which contained municipal and industrial waters as well as surface water runoff.

**Table 2:** Overview of the inflow water quality for mature wetland systems without dilution (preliminarily treated domestic wastewater) for the period from 8 April 2013 to 24 December 2014

Parameter	Unit	Number	Mean	Standard deviation	Minimum	Maximum
Chemical oxygen demand	mg/l	27	285.3	80.33	112.0	385.0
Biochemical oxygen demand	mg/l	43	124.8	84.58	40.0	197.0
Ammonia-nitrogen	mg/l	23	40.2	14.8	3.10	70.0
Nitrate-nitrogen	mg/l	23	1.1	0.9	0.25	4.2
Orthophosphate-phosphorus	mg/l	26	13.3	5.5	3.5	24.1
Suspended solids	mg/l	48	147.8	76.13	28.0	377.0
Turbidity	NTU	43	70.7	47.53	11.6	222.0
pH	–	44	7.72	0.5	6.30	8.40
Dissolved oxygen^a^	mg/l	95	7.1	2.87	0.1	11.4

^a^Start of measurement on 1 May 2014

Five Aqua Medic Titan chillers (A1–A5; Aquacadabra, Barnehurst Road, Bexleyheath, UK) were connected with two tab water storage tanks (S1 and S2) and applied to maintain the combined root system and debris layer of all wetland filters at semi-natural below-surface temperature of approximately 12 °C (Fig. 1). In order to imitate pure diesel fuel spillages, two dosages of diesel fuel were poured out into filters 1, 3 and 5 and into one of the two control filters (control A) on 26 September 2013 and 26 September 2014, respectively (Table 1). The selection of these two diesel dosages is linked to a parallel study testing the long-term performance of vertical flow constructed wetlands treating domestic wastewater subjected to diesel spillages (Al-Isawi et al. 2015a, b). The first low dose of 130 g (equals to an inflow concentration of 20 g/l) diesel was poured into the filters to test the impact of hydrocarbon on the wetlands during 1 year (acclimatisation stage). Thereafter, a high dosage of diesel (975 g; similar to an inflow concentration of 150 g/l) was applied to assess the treatment performance of wetland systems. Diesel fuel samples were purchased from a petrol station owned by Tesco Extra (Pendleton Way, Salford, UK).

The chemical oxygen demand (COD) was applied as a relative measure to distinguish between low and high loads as indicated in Table 1. A preferred inflow COD value estimated at about 280 mg/l (range between 112 and 625 mg/l) was set for wetland filters with a high loading rate (filters 5 and 6). The remaining filters 1, 2, 3, 4, 7 and 8 received domestic wastewater, which has been diluted with dechlorinated tap water. The preferred inflow COD for these filters was about 138 mg/l (range between 43 and 350 mg/l).

### Water quality analysis

The procedure for water quality sampling and the appliances used for water quality parameter measurements were determined according to the American Public Health Association (APHA 2012) and described in detail by a previous study published by Al-Isawi et al. (2015b). Routine water quality sampling of various variables (for sample numbers and frequencies, refer to data illustrations) was conducted to monitor the water quality and examine the performance of the treatment. The spectrophotometer manufactured by HACH Co. model DR2800 (Hach, Loveland, CO, USA) was applied for water quality analysis of COD, NH_4_-N, NO_3_-N, PO_4_-P and suspended solids (SS).

The BOD was estimated with the OxiTop IS 12-6 system, which is a manometric measurement device supplied by the Wissenschaftlich-Technische Werkstätten (WTW, Weilheim, Germany). Nitrification was suppressed by adding 0.05 ml of 5 g/l N-allylthiourea (WTW chemical solution no. NTH600) solution per 50 ml of sample liquid. Turbidity was measured with a Turbicheck Turbidity Meter (Lovibond Water Testing, Tintometer Group, The Tintometer Limited, Lovibond House, Solar Way/Solstice Park, Amesbury, UK). The redox potential was measured with a VARIO pH meter (WTW, Weilheim, Germany), and the electrical conductivity was measured using a Mettler-Toledo AG (Schwerzenbach, Switzerland) conductivity meter. The pH was determined with a sensION+ Benchtop Multi-Parameter Meter (Hach Lange, Düsseldorf, Germany). The dissolved oxygen was obtained using a Hach Lange (Salford, England, UK) HQ30d dissolved oxygen meter.

Total petroleum hydrocarbons (TPHs) were estimated by gas chromatography and flame ionisation by Exova Health Sciences (Hillington Park, Glasgow, UK) according to the “TPH in waters (with aliphatic/aromatic splitting) method” (Exova Health Sciences 2014), which is accredited to the British Standard (BS) method BS EN ISO IEC 17025 by the UK Accreditation Service and compatible to the International Organization for Standardization (ISO) standards (e.g. ISO17025), BS method BS DD 220 1994 and American Standard methods (US Environmental Protection Agency (US EPA) Method 3510C and US EPA SW846 Method 8015).

Nutrients and trace elements for all water samples were measured using a Varian 720-ES Inductively Coupled Plasma–Optical Emission Spectrometer (ICP-OES; Agilent Technologies UK Ltd., Wharfedale Road, Wokingham, Berkshire, UK). The analysis was performed to determine nutrient and trace element concentrations. Glassware bottles were used to preserve 50 ml of each water sample at 4 °C (EPA 1994). The samples were then acidified, if appropriate, by adding 1 ml of 70 % concentrated nitric acid to dissolve any suspended material to extract heavy metals and to reduce the pH to below 2, which was required for analysis. Filter papers with a diameter of 0.45 μm were then used to filter the water samples before analyses by ICP-OES.

The density of the wetland plants, canopy height and the above-ground biomass were monitored for all filters (Table 3). The dead above-ground biomass for each wetland filter was harvested, cut into 2-cm-long pieces, dried and subsequently weighed at the end of each winter. Thereafter, the organic matter was returned to the corresponding wetlands by placing it on top of the litter zone for natural degradation to take its course.

**Table 3:** Growth characteristics of *Phragmites australis* (Cav.) Trin. ex Steud. (Common Reed) for the period between September 2013 and April 2014

Wetland filters	Leave number	Stem number	Mean stem length (standard deviation) (mm)	Stem diameter (standard deviation) (mm)
Filter 1	34	7	908.3 ± (24.33)	2.6 ± (0.92)
Filter 2	53	13	735.5 ± (7.17)	2.5 ± (0.26)
Filter 3	56	9	980.0 ± (18.44)	3.2 ± (0.45)
Filter 4	84	16	887.1 ± (8.74)	3.2 ± (0.67)
Filter 5	14	2	940.0 ± (1.41)	2.6 ± (0.66)
Filter 6	96	17	891.3 ± (9.38)	2.8 ± (0.46)
Filter 7	139	21	913.0 ± (11.62)	3.2 ± (0.50)
Filter 8	224	30	1010.7 ± (15.48)	3.1 ± (0.52)
Control A	0	0	0	0
Control B	16	5	686.0 ± (8.44)	2.4 ± (0.52)

### Chilli quality analysis

At the end of the growth season, trace minerals and potentially poisonous pollutants for chilli fruits were analysed for a randomly selected number of fruits. Chilli plant analysis was performed (Plank 1992) using a dried weight of >0.3 g for digestion. The dried samples were ground to a fine powder in a James Martin ZX809X Spice and Coffee Grinder (WAHL Global, Herne Bay Trade Park, Sea Street, Kent, UK). Samples were turned into white ash in a carbolite muffle furnace at 550 °C for 4 h. The ash samples were dissolved in 7 ml of 70 % concentrated nitric acid. Thereafter, the samples were diluted with deionised water up to 25 ml and transferred into 15-ml polystyrene tubes to be examined by a Varian 720-ES ICP-OES analysis.

### Selected fruiting vegetable and growing of chilli

#### Overview

Chillies were chosen to assess the usability of the vertical constructed wetlands treating domestic wastewater for irrigation purposes. Chilli (De Cayenne; *Capsicum annuum* (Linnaeus) Longum Group ‘De Cayenne’) is a good crop often seen as ideal for growing in greenhouses. This plant is usually easy to grow and cost-effective and has a good nutritional value (Nickels 2012).

The literature indicated that there is no risk of microbiological contamination for chillies, which are not growing in direct contact with soil and/or irrigation wastewater (Cirelli et al. 2012). This is particularly true for the edible parts (Norton-Brandão et al. 2013; Christou et al. 2014).

Chilli (De Cayenne), as part of the verve brand (product code: 362387), was supplied by B&Q plc (Chandlers Ford, Hampshire, England, UK). The chilli planting periods were (a) germination period, (b) first planting after germination period, (c) first replanting period before fruiting, (d) second replanting period after the development of the first set of fruits and (e) second replanting period after fruiting (i.e. second diesel spill on 26 September 2014). The first dose of diesel fuel was added on 26 September 2013 (Al-Isawi et al. 2015a).

#### Growing of chillies: germination stage

In this experiment, 216 seeds were sown in a propagator (verve; B&Q plc) into seed and cutting compost (verve; B&Q plc) and subsequently covered with 6 mm of compost on 12 February 2014. Each propagator contained 72 planting cells with a mean depth of 5 cm (only planted up to about 4 cm; measured before initial watering) and square sides of about 3.5 cm. The compost comprised 58 % sustainably sourced *Sphagnum* (peat moss) and unspecified amounts of components such as composted bark, green compost and wood fibre. Essential nutrients and trace minerals (lasting for approximately 6 weeks) were also part of the product. The remaining 42 % comprised among other components more than 48 % of non-peat composted organic material.

The propagators were located within a dark room. The compost was kept moist until seed germination. Table 4 provides a summary of the environmental conditions for all locations associated with plant growth. In the period of plant germination, the temperature was maintained between 16.5 and 20.2 °C (average of 19.8 °C).

**Table 4:** Overview of environmental boundary conditions associated with the planted chillies

Parameter	Unit	Overall^a^	FPGP^b^	FPAGP^c^	SRPBF^d^	SRPAF^e^	SRPAFD^f^
Temperature (one-off record during greenhouse visit)	°C	18.5 ± 5.35 (188)	19.8 ± 1.92 (19)	26.9 ± 1.30 (18)	18.3 ± 3.03 (19)	20.3 ± 2.81 (85)	11.5 ± 3.49 (47)
Temperature (minimum within a 24-h period)	°C	15.0 ± 4.63 (186)	16.5 ± 2.37(19)	15.7 ± 3.28(16)	14.7 ± 1.86 (19)	17.8 ± 2.80 (85)	9.9 ± 3.57 (47)
Temperature (maximum within a 24-h period)	°C	19.7 ± 5.69 (186)	20.2 ± 4.93(16)	29.7 ± 3.46 (17)	23.1 ± 4.69 (19)	22.7 ± 2.96 (85)	13.0 ± 3.71 (47)
Relative humidity (one-off record during greenhouse visit)	%	77 ± 9.3 (178)	79 ± 5.5(13)	75 ± 6.7(14)	70 ± 6.7 (22)	76 ± 7.4 (82)	84 ± 9.6 (47)
Relative humidity (minimum within a 24-h period)	%	64 ± 15.4 (185)	63 ± 12.1(19)	68 ± 10.7(15)	52 ± 15.5 (22)	62 ± 10.9 (82)	73 ± 17.3 (47)
Relative humidity (maximum within a 24-h period)	%	85 ± 7.5 (185)	84 ± 6.3(18)	87 ± 4.6(16)	77 ± 5.6 (22)	83 ± 4.9 (82)	92 ± 6.7 (47)
Temperature (one-off record outside the greenhouse)	°C	14.5 ± 4.53 (137)	14.9 ± 3.45 (11)	15.3 ± 2.78 (17)	17.2 ± 1.16 (7)	17.4 ± 2.37 (55)	10.7 ± 3.88 (47)
Relative humidity (one-off record outside the greenhouse)	%	53 ± 10.7 (136)	54 ± 11.2(15)	55 ± 12.4(12)	54 ± 10.0 (7)	48 ± 10.9 (55)	56 ± 13.5 (47)

Note that the number of observations for temperature and humidity is given in brackets

^a^12 February 2014 to 24 December 2014

^b^First planting (germination period): 12 February 2014 to 09 March 2014

^c^First replanting (after germination period): 10 March 2014 to 07 April 2014

^d^Second replanting period before fruiting: 08 April 2014 to 11 May 2014

^e^Second replanting period after fruiting (i.e. development of first fruit): 12 May 2014 to 25 September 2014

^f^Second replanting period after fruiting (second diesel spill on 26 September 2014): 26 September 2014 to 24 December 2014

#### Growing of chillies: first planting after germination

Germination of some seeds was noticed on 10 March 2014. All pots were relocated to a lab fitted with OSRAM HQL (MBF-U) High Pressure Mercury Lamp (400 W; Base E40) grow lights provided by OSRAM (North Industrial Road, Foshan, Guangdong, China) and supported by a H4000 Gear Unit, which was supplied by Philips (London Road, Croyden CR9 3QR).

The lights were set on timers, simulating sunrise and sunset times in Greater Manchester. Light was measured using the Lux meter ATP-DT-1300 (TIMSTAR, Road Three Industrial Estate, Winsford, Cheshire, England, UK) for the range between 200 and 50,000 lx. Just above the top of the plants, values between 3855 and 12,316 lx (mean of 6921 lx) were recorded. Humidity and temperature were monitored by a Thermometer Hygrometer Station provided by wetterladen24.de (JM Handelspunkt, Geschwend, Germany). The temperature was controlled using an electrical heater. The humidity was artificially increased by humidifiers (Challenge 3.0-L Ultrasonic Humidifier; Argos, Avebury Boulevard, Central Milton Keynes, England, UK). The observed relative humidity ranged between 68 % (±10.7 %) and 87 % (±4.6 %).

The temperatures above the plants ranged between 15.7 and 29.7 °C (average of 26.9 °C). The propagator covers were kept on top of the corresponding bases (gap of about 6 cm) until the first seedlings reached the covers on 15 March 2014.

#### Growing of chillies: second planting

The second planting of the strongest 90 chilli plants took place when the majority of seedlings had at least two true leaves, which was around 8 April 2014. The remaining weakest 96 chilli plants were not used. Thirty chilli plants either did not germinate or died before replanting. All plants were relocated into the greenhouse (same place where the wetland system is located).

Table 5 outlines the experimental design regarding plant number allocations after replanting in compost covered by bark. Chillies were replanted individually into 10-l round plastic plant pots received by scot plants (Hedgehogs Nursery, Crompton Road, Glenrothes, Scotland, UK). The pot dimensions were 22.0 cm for height, 22.0 cm for the bottom diameter and 28.5 cm for the top diameter. The top 2 cm was not planted. Chillies were planted to a depth of 17.5 cm and covered by 2.5 cm of bark (B&Q verve range) based on mixed wood.

**Table 5:** Experimental design in terms of plant number allocations after replanting in compost covered by bark

Inflow source	Chilli number	Contaminated by diesel	Chemical oxygen demand load	
			Mean	Standard deviation
			g/(m^2^ × day)	g/(m^2^ × day)
Filter 1 outflow	C1; C2; C3; C4; C5; C6	Yes	4.3	2.228
Filter 2 outflow	C7; C8; C9; C10; C11; C12	No	1.1	0.444
Filter 3 outflow	C13; C14; C15; C16; C17; C18	Yes	4.9	2.893
Filter 4 outflow	C19; C20; C21; C22; C23; C24	No	1.2	0.638
Filter 5 outflow	C25; C26; C27; C28; C29; C30	Yes	4.8	3.085
Filter 6 outflow	C31; C32; C33; C34; C35; C36	No	1.4	0.712
Filter 7 outflow	C37; C38; C39; C40; C41; C42	No	0.9	0.380
Filter 8 outflow	C43; C44; C45; C46; C47; C48	No	1.5	1.244
Control A outflow	C49; C50; C51; C52; C53; C54	Yes	3.1	1.492
Control B outflow	C55; C56; C57; C58; C59; C60	No	0.3	0.244
Deionised water	C61; C62; C63; C64; C65; C66	No	0.0	0.000
Tap water (100 %)	C67; C68; C69; C70; C71; C72	No	0.1	0.003
Tap water with fertiliser (0.7 ml/l)	C73; C74; C75; C76; C77; C78	No	0.1	0.003
Wastewater (20 %); tap water (80 %)	C79; C80; C81; C82; C83; C84	No	1.5	0.669
Wastewater (100 %)	C85; C86; C87; C88; C89; C90	No	8.2	2.866

Original seed planting reference numbers; chilli (C1–C90)

Some plants were fed with a liquid fertiliser from the B&Q verve range with nitrogen to phosphorus to potassium ratio of 4:4:4. The TN component was 4 %. Phosphorus pentoxide (P_2_O_5_) and potassium oxide (K_2_O) made up 4 % each.

### Analysis of data

Microsoft Excel and IBM SPSS v22 (IBM Corp. 2013) were used to perform variance analysis between different treatment variables. The Mann-Whitney *U* test was used to test the differences for corresponding water quality parameters. Where appropriate, significant (*p* < 0.05) findings have been highlighted.

## Results and discussion

### Quality analysis for both water used for irrigation and chillies

#### Overview

**Fig. 3:**
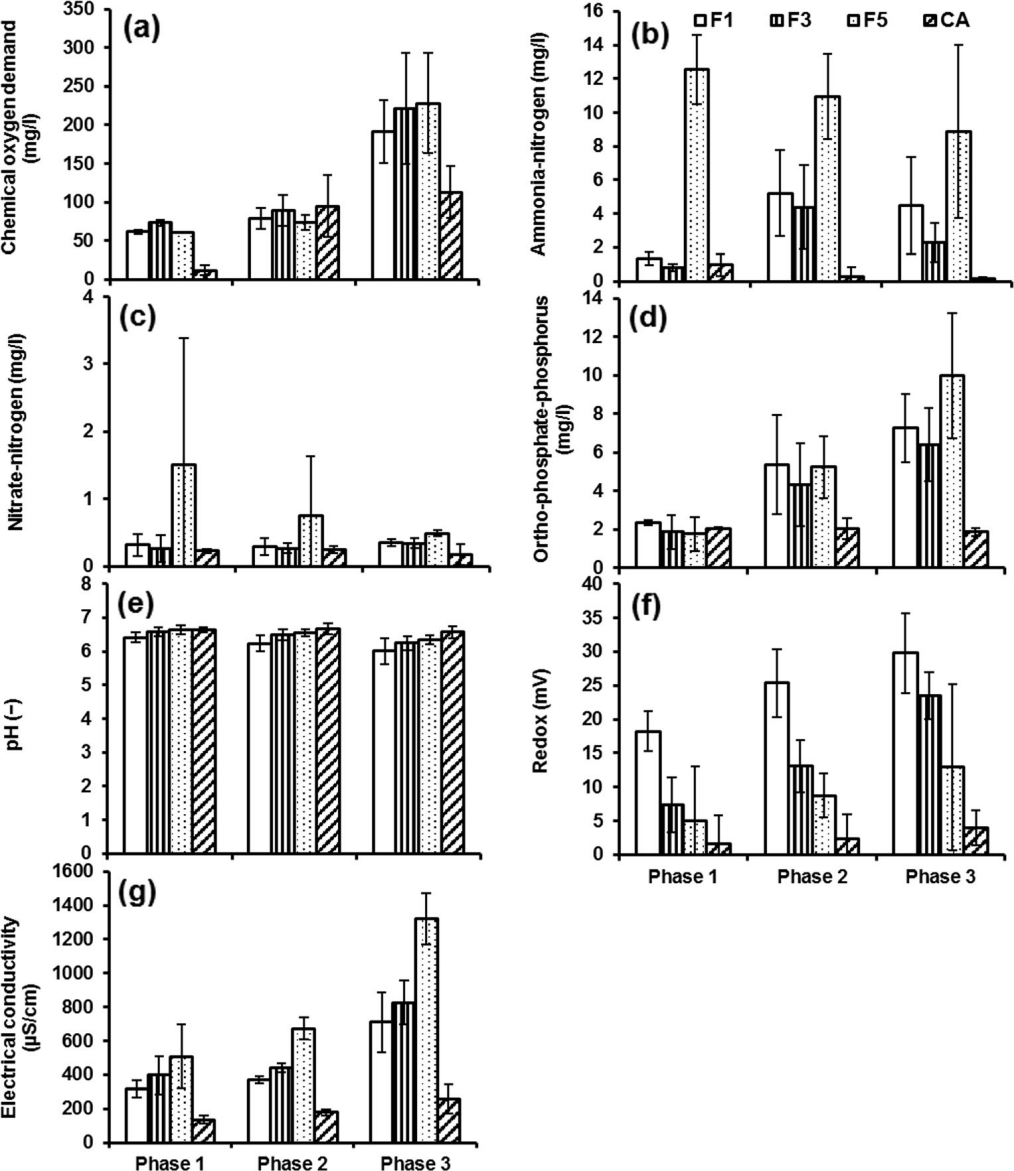
Mean and standard deviation of water quality parameters of irrigation water obtained from wetland filters contaminated with diesel: **a** chemical oxygen demand, **b** ammonia-nitrogen, **c** nitrate-nitrogen, **d** orthophosphate-phosphorus, **e** pH, **f** redox (potential) and **g** electric conductivity. *F1* wetland filter 1, *F3* wetland filter 3, *F5* wetland filter 5, *CA* control A (wetland filter receiving tap water)

**Fig. 4:**
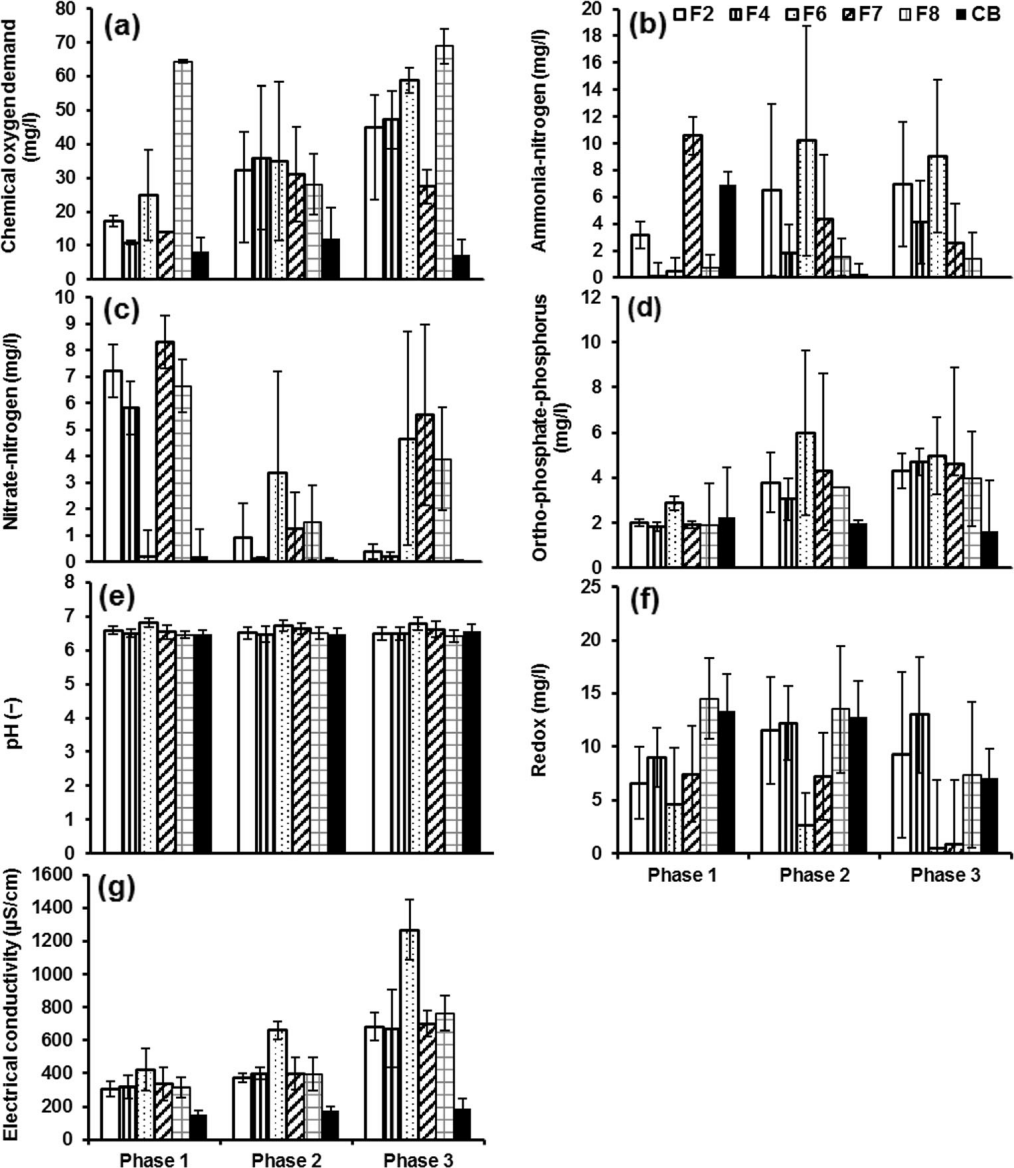
Mean and standard deviation of water quality parameters for irrigation water obtained from wetland filters without diesel contamination: **a** chemical oxygen demand, **b** ammonia-nitrogen, **c** nitrate-nitrogen, **d** orthophosphate-phosphorus, **e** pH, **f** redox (potential) and **g** electric conductivity. *F2* wetland filter 2, *F4* wetland filter 4, *F6* wetland filter 6, *F7* wetland filter 7, *F8* wetland filter 8, *CB* control B (wetland filter receiving tap water)

**Fig. 5:**
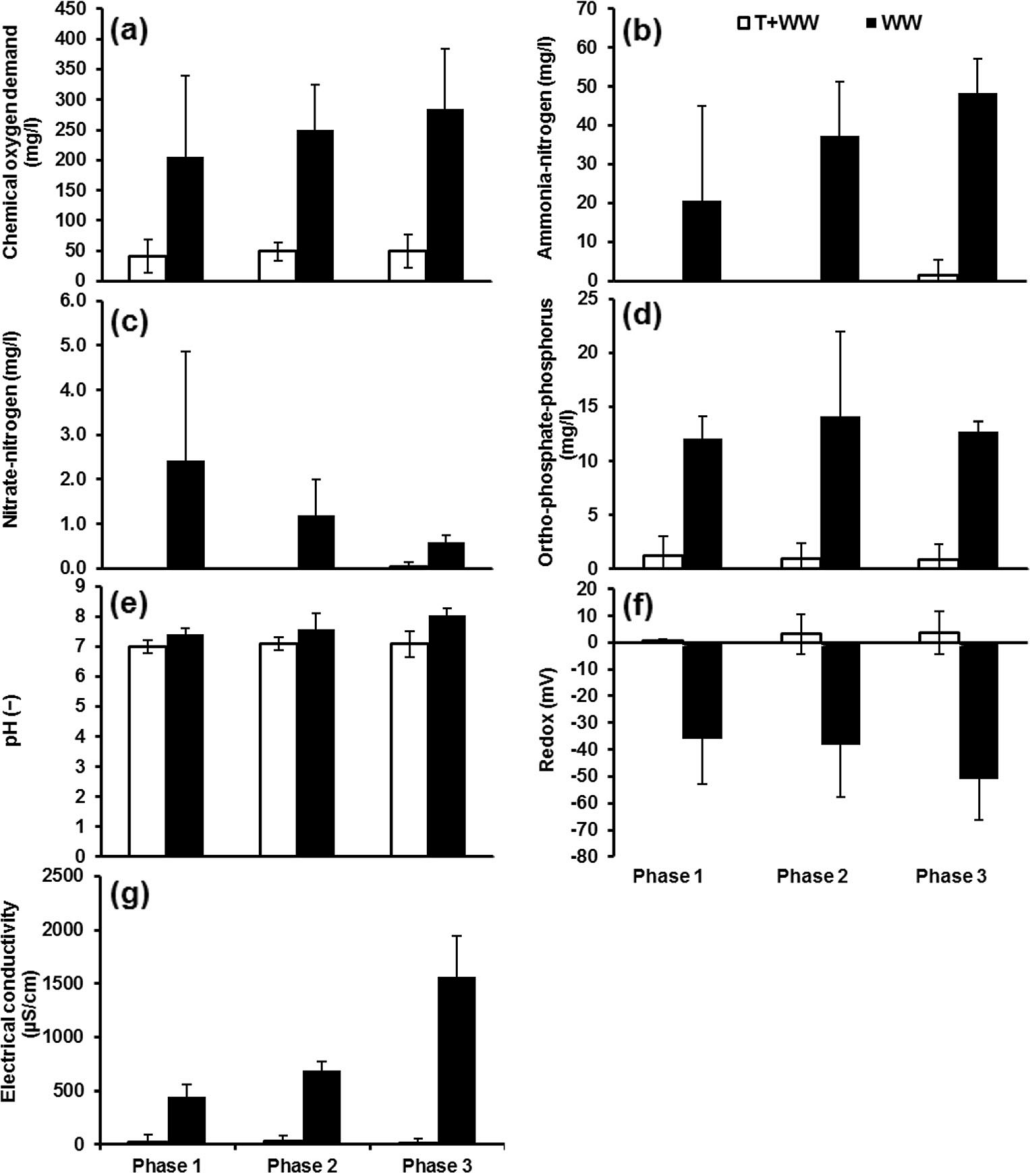
Mean and standard deviation of water quality parameters for various irrigation water types for comparison purposes. *WW + T* one part wastewater mixed with four parts of tap water, *WW* preliminarily treated wastewater

The wetland effluent was used as the influent for the chillies. Figures 3, 4 and 5 indicate the variations of water quality parameters of the irrigation water. The changes in water quality parameters were compared according to three planting phases: phase 1 (planting period before fruiting), phase 2 (planting period after fruiting) and phase 3 (planting period after fruiting and after the second diesel dosage). The water quality parameters of particular focus are COD, NH_4_-N, NO_3_-N, PO_4_-P, pH, redox and electric conductivity.

The filters were grouped into three sets: set 1 for filters subject to contamination with diesel (filters 1, 3 and 5 as well as control A); set 2 for filters without diesel contamination (filters 2, 4, 6, 7 and 8 as well as control B); and set 3 for comparison purposes (preliminary treated wastewater, preliminarily treated wastewater (one part) mixed with tap water (four parts), tap water, deionised water and tap water with fertiliser (0.7 ml/l)). The last three types of irrigation water indicated no notable changes over the period of the experiment and thus are not presented in Fig. 5 (for more details regarding mean, standard deviation and sample number values for all water quality parameters over the three phases, refer to Online Resource 1).

#### Water quality of wetland filters (set 1)

The planting phase 3 showed a notable increase in COD concentration values compared with those in both phases 1 and 2 (Fig. 3a). This could mainly be attributed to the effect of the application of the second dosage of diesel fuel. Hydrocarbon compounds such as diesel are generally linked to high COD values (Scholz 2010, 2015). The lowest value of COD for filter 1 can be assigned to the presence of a substrate of larger aggregate diameter (20 mm), which has been shown to enhance oxygen supply, and better wastewater distribution provides an opportunity to develop a strong layer of biofilm within the voids between aggregates (Sani 2013a). This layer improved with time, as the system started to mature (microbial acclimatisation). An active biofilm increases the biodegradation process during the three periods (Harvey et al. 2002).

The overall mean concentrations of NH_4_-N (Fig. 3b) from low to high followed this order: filter 3 < filter 1 < filter 5. The corresponding values were 3.1, 4.5 and 10.1 mg/l, respectively. The overall mean concentration of control A was estimated at 0.3 mg/l. A significant difference (*p* < 0.05) was noted between NH_4_-N of filter 3 (low inflow load) compared to filter 5 (high inflow load). Significant differences between filters are summarised in Online Resource 2.

The NH_4_-N concentrations for sample water of filter 5 exceeded the corresponding threshold of 5 mg/l (Food and Agricultural Organization (FAO) 2003). This can be attributed to the effect of a high loading rate (concentrated inflow without dilution) as discussed, previously (Al-Isawi et al. 2015a).

As far as NO_3_-N (Fig. 3c) is concerned, the concentrations for filter 3 compared to those of filter 5 were significantly (*p* < 0.05) different from each other. This indicates the impact of the inflow loading rate of wetland systems on outflow water NO_3_-N concentrations (Table 1) as indicated previously (Vymazal 2010; Gajewska et al. 2014; Scholz 2015). The overall mean concentrations followed this order: filter 1 (0.3 mg/l) = filter 3 (0.3 mg/l) < filter 5 (0.7 mg/l). The NO_3_-N mean value for control A was estimated at 0.2 mg/l. The results reveal that NO_3_-N concentrations for all examined wetland outflow waters are less than the maximum threshold, which is 30 mg/l (FAO 2003). The presence of hydrocarbon in the wetland filters results in a reduction of the nitrate concentration in the outflow water (Liu et al. 2011; Al-Baldawi et al. 2015). In general, biodegradation of diesel spills in filters 1, 3 and 5 led to a reduction of the availability of nutrients through these wetland filters. The addition of carbon (via diesel) stimulated the removal of nitrogen, which is needed by microorganisms to decompose hydrocarbons (Liu et al. 2011; Al-Isawi et al. 2015a).

The filters followed the following order for PO_4_-P (Fig. 3d) from low to high: filter 3 < filter 1 < filter 5. The corresponding overall mean concentrations were 5.1, 6.0 and 7.2 mg/l, respectively. The overall mean value of control A was 2.0 mg/l. A threshold value of 2 mg/l has been proposed by FAO 2003 and is considered as a limit for the concentration of orthophosphate-phosphorus. In general, vertical flow constructed wetlands are poor in removing PO_4_-P compounds (Vymazal 2010, 2014; Scholz 2010, 2015), especially when the system reaches maturation (Scholz 2015). Findings revealed that filters 1, 3 and 5 were not able to efficiently remove PO_4_-P. The positive trend refers to the accumulation of PO_4_-P over time (Scholz 2015). The statistical analysis indicated that aggregate diameter as well as contact and rest times did not show any significant (*p* > 0.05) differences between outflow water for these wetland filters.

Regarding pH values (Fig. 3e), the average values from low to high followed this order: filter 1 (6.2) < filter 3 (6.4) < filter 5 (6.5). A slightly higher pH value (6.6) was obtained for control A. For irrigating purposes, pH values for these types of irrigation water were within the normal range between 6.0 and 8.5 (FAO 2008; Scholz 2010).

Regarding the redox potential values (Fig. 3f), the overall mean redox potential values followed this order: filter 5 (9.9 mV) < filter 3 (16.3 mV) < filter 1 (26.2 mV). Control A had a redox potential value of 2.9 mV. The presence of diesel impacted on the rhizosphere and caused a decrease in the redox potential, indicating that the environment is becoming more anaerobic with an increase in diesel (Lin and Mendelssohn 2009; Liu et al. 2011).

Electrical conductivity has been considered as the most important indirect link with salinity, which poses a high threat to crops (FAO 2012). However, the electric conductivity values of all outflow waters were below the maximum threshold of 3000 μS/cm (FAO 2003). A notable increase was recorded for the electric conductivity values of filter 5, particularly during phase 3. Filters 1 and 3 showed a moderate electric conductivity increase over time (Fig. 3g). The overall mean values from low to high were in this order: filter 1 (503 μS/cm) < filter 3 (593 μS/cm) < filter 5 (918.3 μS/cm). As for control A, the value was 205 μS/cm.

Despite the fluctuations and changes observed for all water quality parameters (except for pH) over the three stages, phase 3 showed the most notable changes with significant (*p* < 0.05) increases for most water quality parameters: COD, NO_3_-N, NH_4_-N and total petroleum hydrocarbon (TPH). The corresponding *p* values were 0.000, 0.029, 0.032 and 0.037 in this order. This could mainly be attributed to the impact of the second high dosage of diesel fuel spill (Lin and Mendelssohn 2009; Liu et al. 2011) (for more details about mean, standard deviation and sample number values of water quality parameters over the three phases, see Online Resource 1). The statistical analysis to identify potentially significant differences between filters is summarised in Online Resource 2.

#### Irrigation water from wetland filters without diesel contamination (set 2)

The findings revealed high differences in COD concentrations between the three phases (Fig. 4a). This is due to the seasonal treatment changes of the wetland filters (Tang et al. 2009; Sani et al. 2013b). The overall mean concentrations from low to high were in this order: filter 7 < filter 2 < filter 4 < filter 6 < filter 8. The corresponding values are 27.8, 35.5, 37.5, 43.1 and 48.0 mg/l in this order. Generally, the COD concentrations were remarkably low, if compared to the high inflow COD value (285.3 mg/l) for wetland systems (Table 2), which can be a result of biofilm maturation within the wetland system as microorganisms responsible for biodegradation acclimatise (Scholz 2010; Liu et al. 2011; Sutton et al. 2013). Furthermore, the high COD values of filter 8, if compared with those of the other filters, reflect the impact of low rest time on the treatment performance (Table 1). The importance of long rest time is to aerate the filter substrate and subsequently enhance biodegradation (Scholz 2010, 2015; Vymazal 2010). The data analysis showed no significant (*p* > 0.05) difference in COD values of filter 2 if compared to those for filter 4, indicating no effect of aggregate size on treatment performance (Table 1). However, there is a significant (*p* < 0.05) difference between filters 7 and 8, reflecting the impact of resting time. In comparison, the lowest COD value was recorded for control B (no diesel contamination).

The NH_4_-N concentration data are widely scattered for all three phases (Fig. 4b). This can be explained by the seasonal variations in water quality treatment performance of the wetland systems (Tang et al. 2009; Sani et al. 2013b; Scholz 2015). The overall mean concentrations from low to high were obtained in this order: filter 8 < filter 4 < filter 7 < filter 2 < filter 6. The corresponding NH_4_-N concentrations were 1.4, 2.8, 4.2, 5.2 and 9.2 mg/l, respectively. Control B had a value of 0.5 mg/l. Generally, the outflow NH4-N concentration values were low if compared to the inflow value of 40.2 mg/l. Table 2 highlights the high ability of wetland system to treat NH_4_-N. All NH_4_-N values (except for filter 6) were within the permissible value for crop irrigation of 5 mg/l (FAO 2003).

With respect to NO_3_-N concentrations (Fig. 4c), a decline was observed for filters 2 and 4 (high contact time), whereas control B showed steady concentrations (originally the inflow water without nutrients) over the three examined phases. This could be attributed to a high contact time that resulted in the provision of more time for treatment processes to remove pollutants (Vymazal 2010; Al-Isawi et al. 2015a, b; Scholz 2015). The average NO_3_-N concentrations from low to high were obtained in this order: filter 4 < filter 2 < filter 8 < filter 7 < filter 6. The corresponding concentrations were 0.5, 1.0, 2.9, 3.3 and 3.8 mg/l, respectively. The concentration of control B was 0.1 mg/l. Statistically, there is a significant (*p* < 0.05) difference of outflow NO_3_-N values for filters 4 and 7 highlighting the impact of low contact time on the NO_3_-N treatment performance of wetland systems (Table 1). Moreover, for filter 4, a significant (*p* < 0.05) difference with filter 6 in terms of outflow NO_3_-N values was also noted. This indicates the impact of inflow COD load (Table 1) on the treatment performance of NO_3_-N within the wetland filters (Vymazal 2010; Al-Isawi et al. 2015a, b). In general, all NO_3_-N outflow values were very low (Fig. 4c), which are below the threshold of 30 mg/l (FAO 2003).

With regard to PO_4_-P (Fig. 4d), all filters showed upward tendencies, whereas control B was linked to a slight drop. The overall mean concentrations from low to high followed this order: filter 4 = filter 8 < filter 2 < filter 7 < filter 6. The corresponding values were 3.6, 3.6, 3.8, 4.2 and 5.3 mg/l, respectively. Furthermore, the concentration of control B was 1.9 mg/l. The PO_4_-P concentrations (except for control B) were higher than the threshold limit (2 mg/l) for irrigation use (FAO 2003). This is because of the difficulty to remove accumulated phosphorus particles by constructed wetlands (Vymazal 2010; Scholz 2015).

As for pH (Fig. 4e), despite some fluctuations over the three periods, the majority of the data were around 6.5. The pH values were within the normal range of 6.0 to 8.5 (FAO 2003). Concerning the redox potential values (Fig. 4f), some negative values were measured over the three stages, in particular during phase 3. Filters 2 and 4 remained unchanged, while a decline was noted for the remaining filters.

A remarkable change was recorded for electric conductivity (Fig. 4g). Phase 3 indicated a significant (*p* < 0.05) increase compared to phases 1 and 2. Filter 6, which received a high loading rate compared to those of other filters, was linked to sharp trend reversals. Filters 2, 4, 7 and 8 had similar data trends, whereas control B remained unchanged. However, the electric conductivity for all wetland outflows complied with the threshold of 3000 μS/cm (FAO 2003) (for more details concerning mean, standard deviation and sample number values for all water quality parameters over the three phases, see Online Resource 1). Moreover, significant differences between filters are summarised in Online Resource 2.

#### Standard water types for comparison purposes (set 3)

The water quality variability for preliminarily treated wastewater (raw wastewater) was rather high, indicating the use of highly variable domestic wastewater (Chu et al. 2004; Scholz 2010, 2015). The COD concentration values were the highest for preliminary treated wastewater (257.0 mg/l) followed by wastewater diluted with tap water (48.4 mg/l). Figure 5a presents the mean COD values for both preliminarily treated wastewater and diluted wastewater. Considering NH_4_-N concentrations, the order of overall mean values from low to high was as follows: deionised water (0.0 mg/l) < tap water (0.2 mg/l) < tap water with fertiliser (2.4 mg/l) < wastewater diluted by tap water (8.0 mg/l) < wastewater (40.2 mg/l). Both preliminary treated wastewater and wastewater diluted with tap water (Fig. 5b) showed elevated NH_4_-N concentrations exceeding the threshold of 5 mg/l according to FAO (2003).

With regard to NO_3_-N, low to high overall mean values followed this order: deionised water (0.0 mg/l) < both tap water and wastewater diluted by tap water (0.2 mg/l) < wastewater (1.1 mg/l) < tap water with fertiliser (5.6 mg/l). Here, also NO_3_-N concentration values for all types of irrigation water were less than the permissible values of 30 mg/l (FAO 2008). The pH values showed the following order from low to high: deionised water (6.0) < tap water (6.3) < tap water with fertiliser (6.3) < wastewater diluted by tap water (7.1) < wastewater (7.7). Overall mean values for both preliminarily treated wastewater and diluted wastewater are shown in Fig. 5e. Although the results indicated that all pH values were within the permissible range (6.5–8.5) according to FAO (2008), the preliminarily treated wastewater values were slightly alkaline. Many micronutrients are less available when the water is alkaline according to the World Health Organization (WHO 2006). The overall mean PO_4_-*P* values for wastewater (13.4 mg/l), tap water spiked with fertiliser (4.4 mg/l) and wastewater diluted with tap water (3.0 mg/l) exceeded the permissible value of 2 mg/l for irrigation (FAO 2003). The overall mean electric conductivity concentrations from low to high were in this order: deionised water (4.1 μS/cm) < tap water (77.21 μS/cm) < wastewater diluted by tap water (182.1 μS/cm) < tap water with fertiliser (185.9 μS/cm) < wastewater (965.4 μS/cm). The electric conductivity values for all types of irrigation waters were below the threshold of 3000 μS/cm (FAO 2003). Overall mean values for both preliminarily treated wastewater and diluted wastewater are shown in Fig. 5g (for more details regarding mean, standard deviation and sample number values of water quality parameters over the three phases, the reader may refer to Online Resource 1).

#### Biochemical oxygen demand, suspended solids, turbidity and dissolved oxygen

The highest mean BOD value was for preliminarily treated wastewater (124.9 mg/l) followed by filter 1 (51.5 mg/l), filter 3 (37.4 mg/l), filter 5 (38.7 mg/l) and diluted wastewater (28.4 mg/l). The higher BOD values observed for filter 1 explain the effect of large aggregate diameter (Table 1). These findings are consistent with those reported by Al-Isawi et al. (2015a, b).

Suspended solids were highest for preliminarily treated wastewater (146.2 mg/l) and preliminarily treated wastewater diluted with tap water (49.0 mg/l) followed by wetlands contaminated with diesel: filter 1 < filter 3 < filter 5. The values of SS for filters contaminated with hydrocarbon are relatively high, if compared with those for filters without hydrocarbon contamination. Filters subjected to diesel spills showed elevated SS concentrations. Initially, dying above-ground *P. australis* plants and decaying biomass contributed to SS and turbidity as by-products of the biodegradation process (De Biase et al. 2011; Scholz 2015). Thereafter, degraded diesel led to additional SS loads as discussed in a related paper on modelling filter clogging by SS (Al-Isawi et al. 2015b).

Similar trends have been noted for turbidity. High values of SS and turbidity enhance the development of hydrophobicity in the soils and, thereafter, impact on plant growth (Chu et al. 2004).

Dissolved oxygen is an important parameter for growing crops. High DO concentrations in irrigation water used for greenhouses can pay huge dividends for growers. Nutrient absorption occurs in the root zone of plants, and it cannot occur unless oxygen is present (Asano 2007). The benefits of dissolved oxygen go beyond mere root growth. Augmented oxygen can lessen root problems such as those associated with pythium and phytophera and can decrease secondary infections (WHO 2006). Higher DO values were generally observed for wetland filters without diesel contamination (2.0–2.9 mg/l) as compared with those for diesel-contaminated filters (1.5–1.7 mg/l). The reduction of the amount of available DO in the diesel-contaminated filters was linked with an improvement in the hydrocarbon removal efficiencies as microorganisms, which are responsible for biodegradation, acclimatised (Sutton 2013). Considering that the concentration of DO in filters is limited, most hydrocarbons are effectively removed through microbial degradation under anoxic and anaerobic conditions (Vymazal 2010) (for more details about mean, standard deviation and sample number values of water quality parameters for the three phases, readers may refer to Online Resource 1).

#### Findings regarding trace elements and potential pollutants

**Fig. 6:**
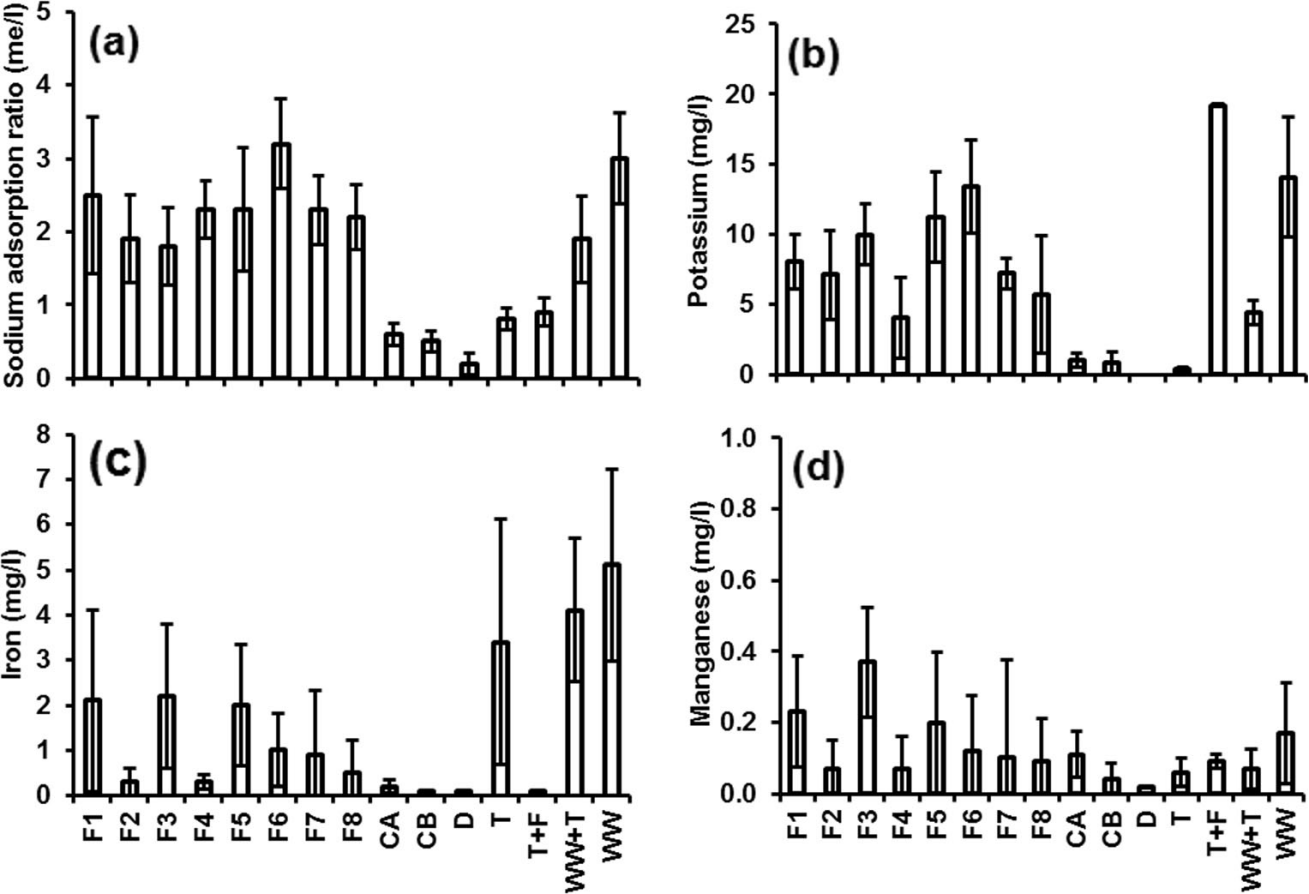
Overview of the inductively coupled plasma (ICP)–optical emission spectrometer findings for selected elements of the inflow waters received by the chilli plants: **a** sodium adsorption ratio (sodium / ((calcium + magnesium) / 2)0.5), **b** potassium, **c** iron and **d** manganese. Elements not shown were not detected; sample number was 15 for data entries. *F1* wetland filter 1, *F2* wetland filter 2, *F3* wetland filter 3, *F4* wetland filter 4, *F5* wetland filter 5, *F6* wetland filter 6, *F7* wetland filter 7, *F8* wetland filter 8, *CA* and *CB*, controls A and B (wetland filters receiving tap water), *D* deionised water, *T* tap water, *T + F* tap water mixed with fertiliser (0.7 ml/l), *WW + T* one part wastewater mixed with four parts of tap water, *WW* preliminarily treated wastewater

Figure 6 provides an overview of the ICP-OES results for selected trace elements measured in the irrigation water. The sodium adsorption ratio (SAR) is defined as the tendency of water to lead to a replacement of calcium (Ca) and magnesium (Mg) ions adsorbed to the soil minerals with sodium (Na) ions (APHA 2012). This indicator is applied to determine the sodium hazard of irrigation water. The findings of the analysed water samples showed that all types of irrigation water have low SAR values between 0.2 and 3.2 me/l (Fig. 6a), which presents no irrigation challenge as the standard range is between 0 and 15 me/l. The pretreated wastewater is therefore suitable for irrigation of edible crops (FAO 2012; Ben Fredj et al. 2013; Tsado et al. 2014).

Considering the FAO (2003) threshold of 2 mg/l for potassium (FAO 2003), the outflows from all wetland filters (except for controls A and B), preliminary treated wastewater and diluted wastewater are linked to relatively high potassium concentrations (Fig. 6b). With regard to manganese (Fig. 6d), the FAO (2003) threshold is 0.2 mg/l. The diesel-contaminated filters 1, 3 and 5 showed high manganese concentrations (Fig. 6d). Manganese represents an essential trace element for growing of crops (SEPA 2005; Almuktar et al. 2015). However, high manganese concentrations are often toxic. Manganese phytotoxicity causes a reduction of biomass and photosynthesis, as well as biochemical challenges including oxidative stress (Millaleo et al. 2010). Regarding iron (Fig. 6c), diesel-contaminated filters were generally relatively high in iron concentrations if compared with those corresponding uncontaminated filters, explaining the impact of diesel contamination on iron concentrations of the outflow waters. However, iron concentrations in all types of irrigated water (with exception to preliminarily treated wastewater) were below the permissible limit of 5 mg/l (FAO 2003; Norton-Brandão et al. 2013).

**Fig. 7:**
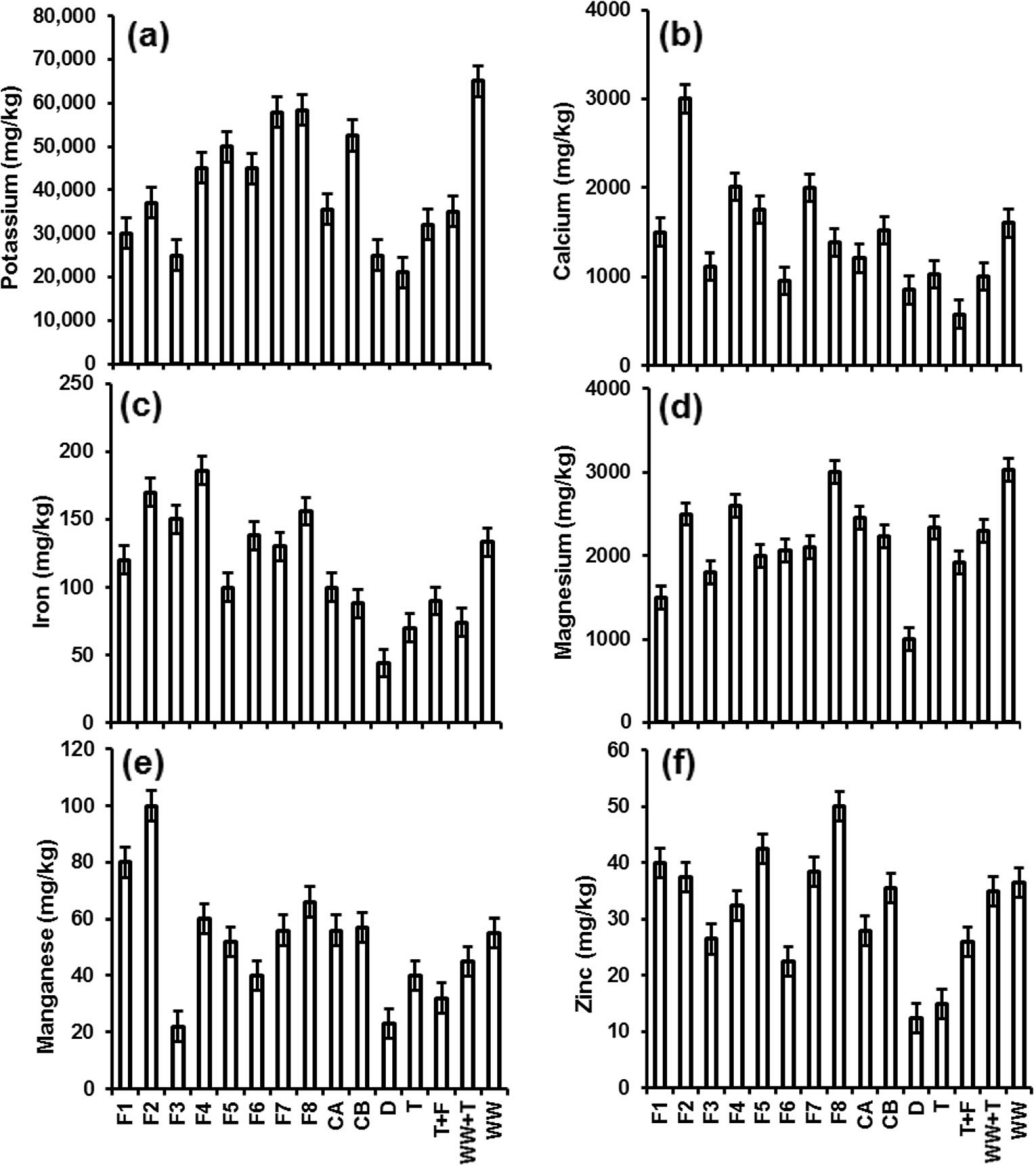
Inductively coupled plasma (ICP)–optical emission spectrometer analysis for selected elements in chilli fruits. Twelfth fruit samples per type of irrigation water were analysed. Elements including arsenic, boron, barium, bismuth, cadmium, cobalt, chromium, copper, lithium, nickel, lead, strontium and titanium were below the detection limits

Figure 7 shows the element concentrations detected in chilli fruits. Elements including arsenic, boron, barium, bismuth, cadmium, cobalt, chromium, copper, lithium, nickel, lead, strontium and titanium were below detection limits. Overall, potassium, calcium and magnesium concentrations in all analysed fruits were higher than those reported by Ciju (2003). Each 100 g of dried chillies contained 1870 mg potassium, 45 mg calcium and 88 mg magnesium. These minerals are important for humans to maintain bone structure, muscle and nerve function control and blood stream. Concentrations for potentially poisonous elements within chillies were all (except for zinc regarding filter 8 on occasions) below recommended world and European thresholds. FAO/WHO (2001) recommended the following thresholds, for example, metals in vegetables: cadmium (0.1 mg/kg), cobalt (50 mg/kg), chromium (2.3 mg/kg), copper (73.3 mg/kg), iron (425 mg/kg), manganese (500 mg/kg), nickel (66.9 mg/kg), lead (0.3 mg/kg) and zinc (100 mg/kg). In addition, EC (2001) has set maximum levels for certain pollutants in food products: copper (20 mg/kg), lead (0.3 mg/kg), zinc (50 mg/kg) and cadmium (0.05 mg/kg).

#### Hydrocarbon analysis findings

**Fig. 8:**
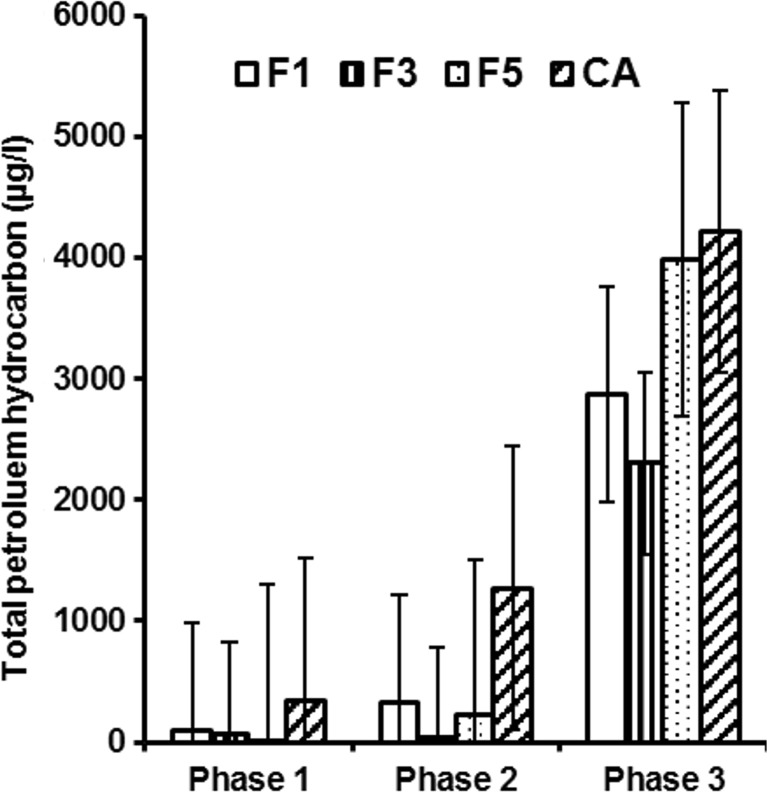
Variation of hydrocarbon concentrations for filters 1, 3 and 5 as well as control A for three planting phases. *F1* wetland filter 1, *F3* wetland filter 3, *F5* wetland filter 5, *CA* control A (wetland filter receiving tap water)

Total petroleum hydrocarbon (TPH) was used to measure the overall hydrocarbon compounds in water samples. Figure 8 provides an overview of TPH values for set 1 wetland filters (contaminated with diesel). Diesel background concentrations were low in the raw wastewater. In the three planting phases, there was a notable reduction in TPH values compared with the high amount of the two inflow diesel spills (20 and 150 g/l). However, variations in TPH concentration values were recorded in the outflow water of filters 1, 3 and 5 as well as control A over three planting phases. Regarding the first and second planting phases (phases 1 and 2), which were during the first diesel spill (20 g/l) period, the TPH concentrations for the outflows from all wetlands except for control A were in compliance, for example, with the Chinese standard for irrigation water quality (State Environmental Protection Administration (SEPA) 2005) of chillies, setting a maximum allowable value of 1 mg/l. The Chinese standards have been referenced here, considering that China is estimated to produce more than 50 % of peppers in the world. While in the third planting phase (during the second diesel spill of 150 mg/l), all wetland filters showed relatively high TPH concentrations in their outflow waters. The TPH concentrations from high to low followed this order: control A > filter 5 > filter 1 > filter 3. However, Fig. 8 shows high values of hydrocarbon concentrations during the second diesel spill period (phase 3). These concentrations are only linked to the one-off high hydrocarbon concentration value resulting from the diesel poured into the wetland systems on 26 September 2014. The corresponding concentrations of hydrocarbon in the effluent gradually decreased over time, reflecting the high treatment performance of the mature wetland systems.

The hydrocarbon reduction in the outflow waters obtained from wetlands subjected to diesel spills is consistent with the increased availability of oxygen (due to tidal flow) in the upper filter location and the subsequent decrease in concentration with depth. Biodegradation of diesel in filters 1, 3 and 5 reduced nutrients. This has been noted for the NH_4_-N and NO_3_-N outflow concentrations (Figs. 3b, c). However, as biodegradation of diesel progressed, it can be assumed that small amounts of remaining hydrocarbon actually enhance the growth of some microorganisms increasing the degradation rate (Liu et al. 2011).

The indirect aeration of aggregates to enhance biodegradation provides root exudates for microbial co-metabolisation of oil (Lin and Mendelssohn 2009). Co-metabolism by microorganisms in the context of this paper can be defined as the simultaneous degradation of two compounds, in which the degradation of root exudates depends on the presence of diesel.

Control A lacking mature biomass (Table 3) exhibited a high TPH concentration over time. Furthermore, *P. australis* (wetland plant) had a delayed and reduced growth rate during the second diesel spill. This is due to diesel toxicity to organisms and macrophytes (Liu et al. 2011; Vymazal 2014; Scholz 2015).

### Growth comparisons of chilli plants

#### Boundary conditions and water consumption

During the three planting phases, the light intensity measurements inside the greenhouse were within the suggested allowable range from roughly 8600 to 17,200 lx (Deli and Tiessen 1969). Flower inhibition and/or abscission (here the natural detachment of flowers) as well as plant growth disorders can be caused if no sufficient light intensity is applied during the growth of plants. These findings are in agreement with what was previously presented in the literature (Almuktar et al. 2015). Table 4 summarises the environmental conditions for all planting periods. According to Nickels (2012), temperatures were within the preferred ranges for various chilli plant growth stages. However, for this experiment and during the period of fruiting (during summer), the temperature records were relatively high during some days between 20 and 29 °C. These temperature records complied with the values linked to the highest photosynthesis rate, which takes place between 24 and 29 °C (Bhatt and Srinivasa 1989). Relative humidity measurements within the range of 60 to 90 % had little impact on plants. Less than 50 % relative humidity could negatively impact the pollination of flowers and the fruit development (Nickels 2012).

Table 6 shows the total water volumes for all plants for various planting stages. The germination period was excluded as all plants during this period were sprayed with tap water. The productivity of plants was independent of water consumption (see Online Resource 3 for more details regarding volume of water consumption for each chilli plant).

**Table 6:** Overview of the total water volumes for chilli plants for different planting periods

Inflow source	Total irrigation water volume^a^ (l)				Standard deviation (l)			
	FRP^b^	SRPBF^c^	SRPAF^d^	SRPAFD^e^	FRP^b^	SRPBF^c^	SRPAF^d^	SRPAFD^e^
Filter 1 outflow	0.25	2.35	14.74	5.57	0.05	0.26	0.27	0.24
Filter 2 outflow	0.25	2.55	17.34	6.48	0.05	0.17	0.54	0.28
Filter 3 outflow	0.25	2.40	16.33	5.51	0.05	0.39	0.35	0.34
Filter 4 outflow	0.24	2.45	17.44	6.28	0.04	0.03	0.27	0.14
Filter 5 outflow	0.25	2.68	17.49	6.33	0.04	0.15	0.40	0.28
Filter 6 outflow	0.26	2.75	18.02	6.56	0.07	0.26	0.83	0.64
Filter 7 outflow	0.26	2.63	18.04	6.59	0.07	0.25	0.30	0.34
Filter 8 outflow	0.25	2.71	17.16	6.86	0.05	0.31	0.69	0.24
Control A outflow	0.25	2.37	15.05	5.80	0.05	0.40	0.33	0.14
Control B outflow	0.25	2.22	16.21	6.23	0.08	0.25	0.90	0.05
Deionised water	0.31	2.60	16.12	5.55	0.08	0.10	0.32	0.11
Tap water (100 %)	0.31	2.60	17.12	6.38	0.07	0.12	0.41	0.28
Tap water with fertiliser (0.7 ml/l)	0.30	3.02	17.60	6.62	0.07	0.04	0.57	0.11
Wastewater (20 %); tap water (80 %)	0.32	2.79	17.83	7.73	0.08	0.25	0.40	0.22
Wastewater (100 %)	0.31	2.80	17.91	7.23	0.07	0.10	0.27	0.18

^a^Each value represents the means of the total water volume based on six replicates

^b^First replanting period: 12 February 2014 to 07 April 2014

^c^Second replanting period before fruiting: 08 April 2014 to 11 May 2014

^d^Second replanting period after fruiting: 12 May 2014 to 25 September 2014

^e^Second replanting period after fruiting (second diesel spill on 26 September 2014): 26 September 2014 to 24 December 2014

#### Wetland design and operation variable impacts on chillies

**Fig. 9:**
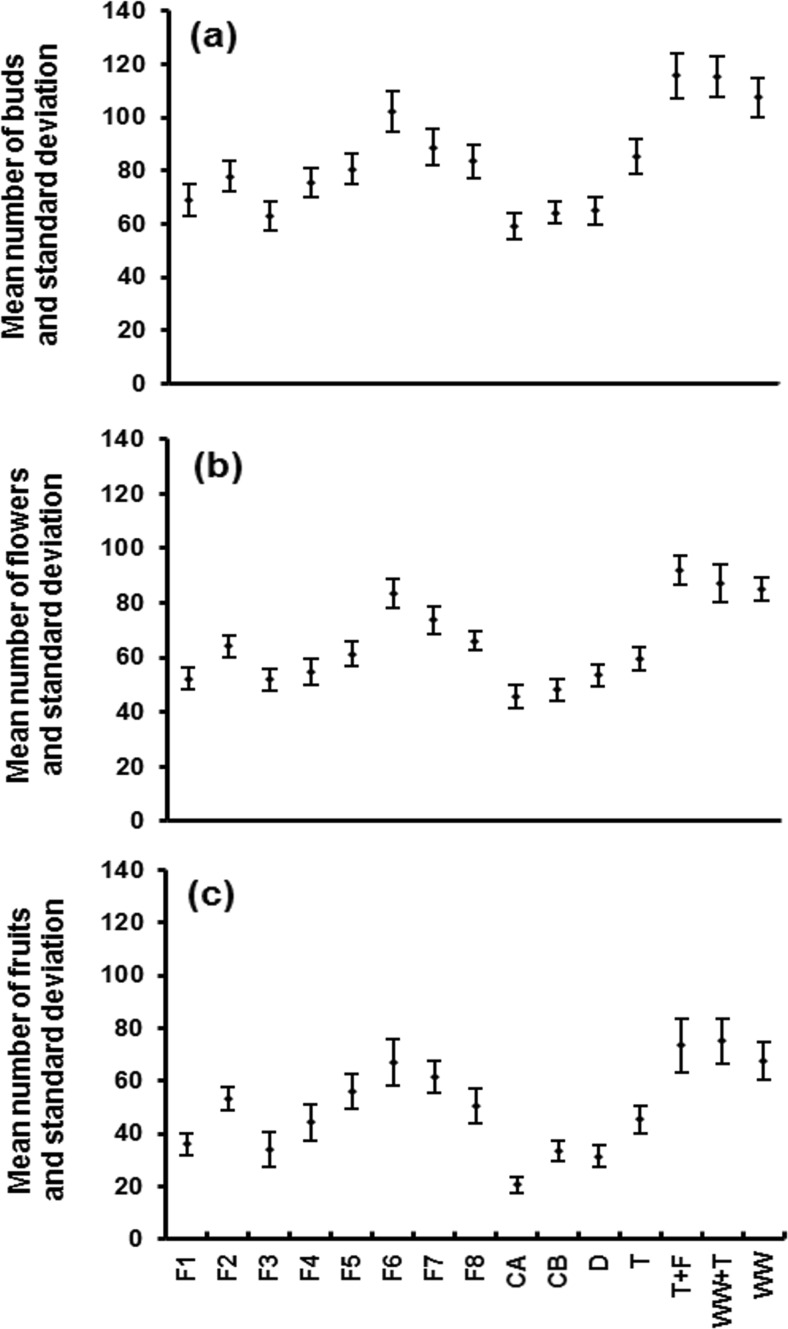
Mean and standard deviation of **a** bud, **b** flower and **c** fruit developments for chilli plants. *F1* wetland filter 1, *F2* wetland filter 2, *F3* wetland filter 3, *F4* wetland filter 4, *F5* wetland filter 5, *F6* wetland filter 6, *F7* wetland filter 7, *F8* wetland filter 8, *CA* and *CB* controls A and B (wetland filters receiving tap water), *D* deionised water, *T* tap water, *T + F* tap water mixed with fertiliser (0.7 ml/l), *WW + T* one part wastewater mixed with four parts of tap water, *WW* preliminarily treated wastewater

The impact of wetland design and operation variables on chilli growth is shown in Fig. 9. In general, the quantity of diesel compounds determines the toxicity to plants and hampers their growth (Singh et al. 2012). Furthermore, accumulated compounds of hydrocarbon in soil media hinder air diffusion through the pores, which causes anaerobic conditions and subsequently permeability reductions of the soil environment, negatively affecting the diversity of microorganisms (Sutton et al. 2013) and thus preventing chilli plants from the uptake of nutrients. In the first and second planting phases (period of first diesel dosage), findings showed no significant (*p* > 0.05) difference in terms of the impact of irrigation water on plant growth between filters with and without diesel contamination. This suggests that small amounts of hydrocarbon would not affect the growth of plants (Al-Baldawi 2015). Moreover, the composition of compost was still fresh without contaminants accumulating at the beginning of the bud development period. After that, with passing time, plant buds were varied highlighting the effect of hydrocarbon accumulation on those pots receiving irrigation water contaminated with diesel (Fig. 9a). Moreover, most flowers were lost in plants irrigated with diesel-contaminated filters illustrating the effect of toxic hydrocarbon compounds (Fig. 9b), particularly during the third planting phase after the second diesel spill (i.e. 3 weeks after application). With regard to the fruits during the third phase, they either fell off or showed reductions in their growth due to the toxicity of chemicals associated with high hydrocarbon compounds (Liu et al. 2011; García-Delgado et al. 2012). Online Resource 4 shows the number of buds, flowers and fruits associated with each chilli plant. Statistically, plants exhibited significantly (*p* < 0.05) fewer fruit numbers than those for plants irrigated with outflows from filters without diesel contamination. For diesel-contaminated filters, small aggregate sizes (filter 3 and 5) performed significantly (*p* < 0.05) better compared to larger sizes (filter 1). Small aggregate diameters correlated positively with large surface areas, allowing more microorganisms to degrade hydrocarbon pollutants (Scholz 2015).

The wetland filter with a high loading rate (filter 5) released more nutrients associated with its effluent compared to filters 1 and 3. Filter 5 received a high inflow load containing high amounts of nutrients (treatment efficiency decreased with increasing nutrient load) compared to filter 3 receiving fewer nutrients, because the influent wastewater was diluted with tap water (Al-Isawi et al. 2015a, b). Filters 1 and 3 suffered from a deficiency in nutrients in the outflow water. This was a result of the impact of hydrocarbon compound degradation processes by microorganisms, which used these nutrients during hydrocarbon biodegradation in the wetland systems (De Biase et al. 2011; Liu et al. 2011). The NO_3_-N values were lower for filters contaminated with diesel compared to those without diesel. The addition of diesel-related carbon enhanced the nitrogen removal by microorganisms (Liu et al. 2011).

The analysis indicated a significant (*p* < 0.037) difference in the fruit numbers between filters 3 and 5; it can clearly be seen that the productivity of fruits associated with filter 5 (56) is better than that associated with filters 1 and 3 (36 and 34, respectively) as shown in Fig. 9c. This can be explained by the continuous supply of nutrients associated with the treated water from this filter, as it receives concentrated domestic wastewater without dilution (Becerra-Castro et al. 2015). Control A (contaminated with diesel) showed the least fruit numbers if compared to other filters. This is due to the lack of nutrients associated with its effluent (Aiello et al. 2007). More explanations of differences between filters can be found in Online Resource 5 summarising the statistical analysis (non-parametric Mann-Whitney *U* test) showing the differences in chilli fruits due to various types of irrigation water.

The time for filling and emptying the filters made a significant (*p* < 0.05) difference on chilli growth in terms of the length, width and weight of fruits. Filters with a short contact time (filter 7) performed better for most water quality parameters, resulting in a good harvest of chilli fruits (62) compared to those with longer contact times (filter 4 linked to 44 fruits). Furthermore, results indicated that there is a significant (*p* < 0.05) difference in terms of rest time (filters 7 and 8). A low rest time means a high frequency of loading the wetland (filter 8), which increases the pollutants associated with this filter (Al-Isawi et al. 2015a; Belhaj et al. 2015). Findings also revealed that good productivity of fruit numbers was associated with a high loading rate (filter 6). This is due to the good performance of water quality outflow parameters and sufficient nutrients for plant growth (Nickels 2012; Becerra-Castro et al. 2015).

The chilli fruit number linked to tap water (45) was less than the one for plants linked to diluted wastewater (75) as shown in Fig. 6c. As the compost becomes depleted of nutrients, the harvest increased for plants receiving pretreated wastewater compared to those plants, which only depend on nutrients received from compost. Furthermore, findings designate that nutrients obtained by chillies due to a combination of wastewater and tap water were too high to result in a profitable harvest (Norton-Brandão et al. 2013) as compared with those linked to preliminary wastewater (66). The high amount of turbidity and SS associated with preliminarily treated wastewater enhances the development of hydrophobicity in soils, which subsequently affected the growth of plants (Travis et al. 2010; Becerra-Castro et al. 2015).

The numbers of buds, flowers and fruits that were obtained in this study markedly differed from those in a related previous study (Almuktar et al. 2015), indicating that greenhouse conditions benefit chilli plant growth over a laboratory environment supported by artificial growth light. Both high temperature and sun intensity during summer resulted in an increase of the chilli yield in the greenhouse environment.

### Cost-benefit analysis

The classification scheme proposed by Almuktar et al. (2015) for laboratory-gown chillies was adopted in this related study (Online Resource 6), which, however, relates to chillies grown in a greenhouse. This study should therefore be of more international interest.

Note that only the variables length, width, weight and bending (Almuktar et al. 2015) were used for classifying the harvested fruits. The economic value of the harvest of chilli plants was estimated according to the mean national prices on the UK market between January and June 2015.

**Fig. 10:**
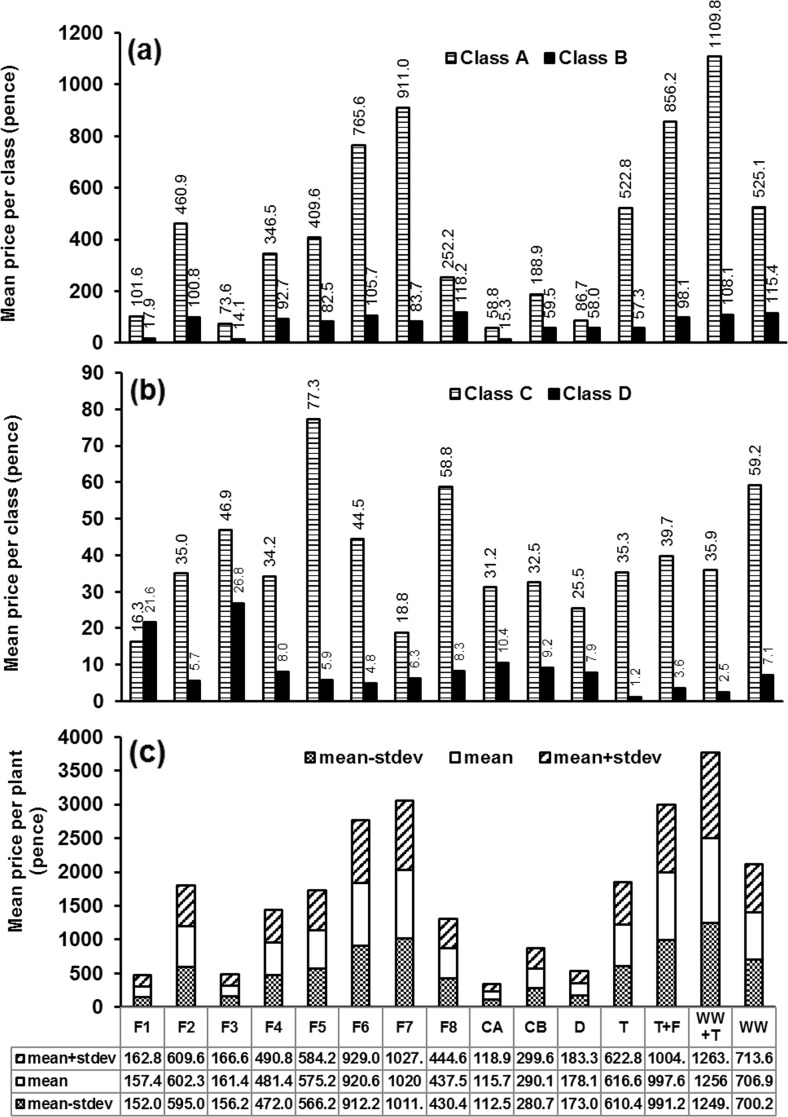
Economic return for varied classes of harvested chilli fruits. No financial return for class E. *F1* wetland filter 1, *F2* wetland filter 2, *F3* wetland filter 3, *F4* wetland filter 4, *F5* wetland filter 5, *F6* wetland filter 6, *F7* wetland filter 7, *F8* wetland filter 8, *CA* and *CB* controls A and B (wetland filters receiving tap water), *D* deionised water, *T* tap water, *T + F* tap water mixed with fertiliser (0.7 ml/l), *WW + T* one part wastewater mixed with four parts of tap water, *WW* preliminarily treated wastewater

Figure 10 indicates the fiscal value of the harvest. Figure 10a, b shows the average price of fruits linked to classes A, B, C and D for each type of irrigation water. However, class E (representing essentially organic waste) has been excluded because no monetary value for fruits is linked to this category. Figure 10c shows the total price for each plant irrigated with one type of water (for more details about the price associated with each chilli plant, see Online Resource 7). The highest average price of harvested fruits, which is estimated at 1256 pence, and the greatest number of fruits of class A were obtained from chillies watered with tap water diluted by wastewater.

The findings concerning the economic return from chilli fruits are not in agreement with those by Almuktar et al. (2015), indicating that greenhouse conditions are better than artificial light growth environments. The average yield price per plant obtained from this study ranged from 300 to 4000 pence, which is significantly (*p* < 0.05) higher than the range from 0 to 150 pence linked to the study by Almuktar et al. (2015).

**Fig. 11:**
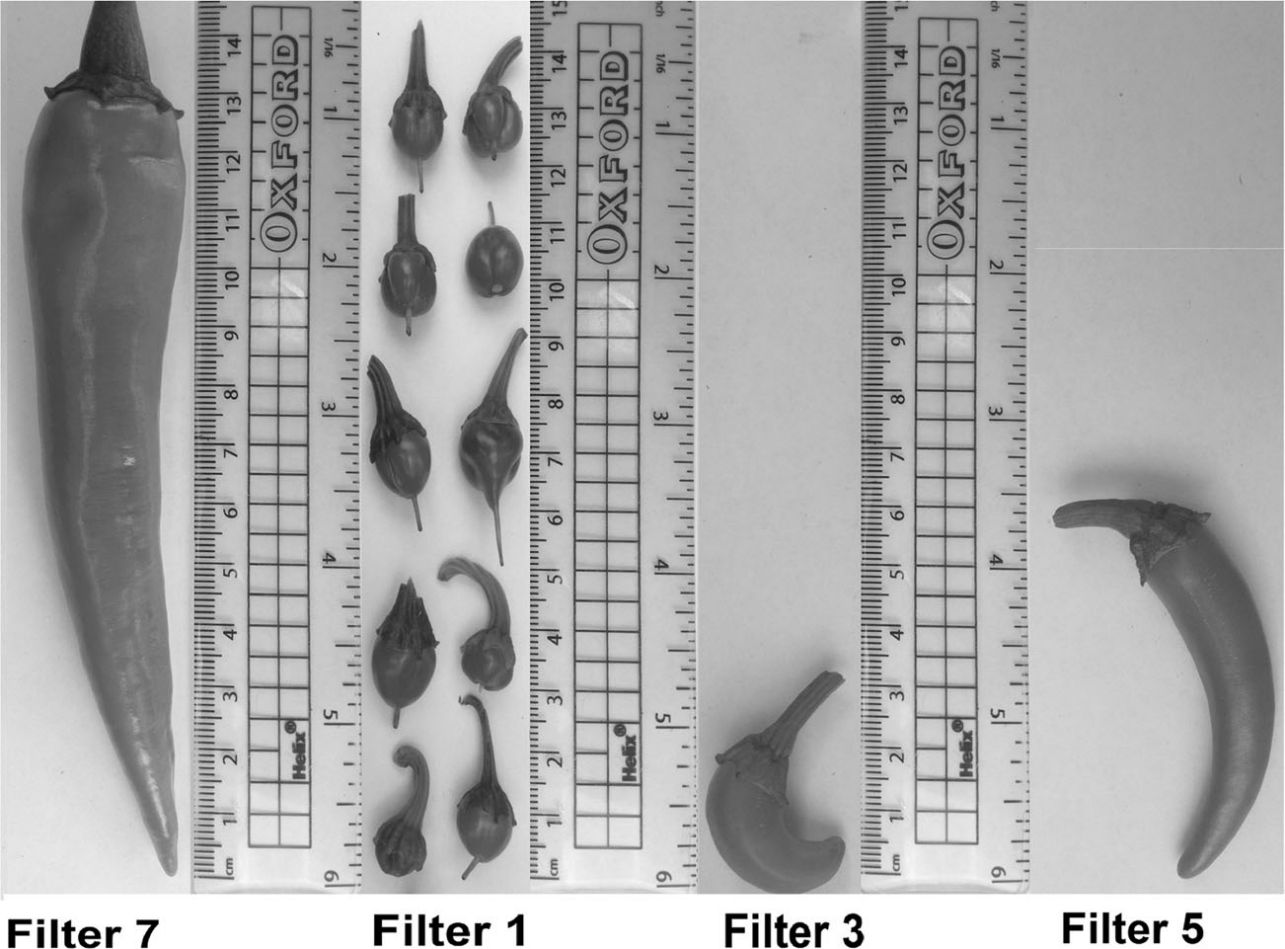
Photographs (taken by Rawaa Al-Isawi) of sample chilli harvests linked to filter 7 (without diesel contamination) and filters 1, 3 and 5 (diesel-contaminated). Unhealthy fruits were associated with outflow waters from filters 1 and 3. *F1* wetland filter 1, *F3* wetland filter 3, *F5* wetland filter 5, *F7* wetland filter 7

For all wetland-based experiments, filter 7 is associated with the greatest yield in terms of its overall economic value. Furthermore, filter 7 provides the highest financial return linked to class A. This can be explained by a combination of small aggregate size, low contact time and high rest time. This interpretation is concordant with what was indicated by Almuktar et al. (2015). Despite that, the irrigation with wastewater diluted by tap water resulted in a higher overall yield (Fig. 10c). Generally, all fruits harvested from diesel-contaminated filters (filters 1, 3 and 5 as well as control A) were weak, indicating the negative impact of diesel contamination on chilli plants. However, filter 5 had the highest number of fruits linked to class A. This is possibly due to the balanced presence of minerals and nutrients that were needed for plant growth due to a high loading rate. Figure 11 shows the growth comparison for the selected fruits harvested from filter 7 (without diesel contamination) and diesel-contaminated wetlands (filters 1, 3 and 5).

The lowest price estimated at 157 pence was associated with filter 1. The fruits linked to filter 3 were slightly better than those associated with filter 1. Most fruits were categorised as classes D and E, which can be explained by the acidic nature of the outflow water from this filter, resulting in a lack of trace elements essential for plant growth (FAO 2008). The plants that were watered with deionised water and control B outflow exhibited a decline in their productivity over time. This could be assigned to nutrient depletion over time (Nickels 2012). For filters without diesel contamination, a high value of fruits was associated with a low contact time (filter 7; 1020 pence) as shown in Fig. 10c. However, the plants associated with filter 6 show a high overall fruit price (921 pence). A significant (*p* < 0.05) number of fruits were linked to class C. The impact of the presence of hydrocarbon on the treatments in terms of price associated with yields was statistically insignificant. However, marketable yields were higher for filters without hydrocarbon contamination.

The outweigh for filters without hydrocarbon to those subject to hydrocarbon influence in terms of mean price per plant (%) was obtained using Eq. 1 as shown in the following:

1 $$ W\left(\%\right)=\left[1-\left(\frac{F_{i\left(\mathrm{with}\ \mathrm{hydrocarbon}\right)}}{F_{i\left(\mathrm{with}\mathrm{out}\ \mathrm{hydrocarbon}\right)}}\right)\right]\times 100 $$

where *W* represents the weight of filters without hydrocarbon to those linked with hydrocarbon in terms of mean price per plant (%). The *W*(%) values for the ratios *filter*_*1*_/*filter*_*2*_, *filter*_*3*_/*filter*_*4*_, *filter*_*5*_/*filter*_*6*_ and *control*_*A*_/*control*_*B*_ were 74, 67, 38 and 60 %, respectively. The overarching performance of filters lacking hydrocarbon to those associated with hydrocarbon is estimated at about 60 %. Results also show that the *W* value for filter_5_/filter_6_ was the lowest among others. It follows that even with a notable adverse impact of diesel contamination, filter 5 performed slightly better than would have been expected. This result is comparable to that published by Singh et al. (2012). However, such results need further investigations to achieve a higher level of performance compared to those without the influence of hydrocarbon.

## Conclusions and recommendations

This paper highlights for the first time the optimum environmental conditions for effective growth of the sample fruiting vegetable chilli in greenhouses using urban wastewater pretreated by mature vertical flow wetlands. An encouraging solution has been successfully proposed to effectively treat and subsequently reuse domestic wastewater in a more sustainable manner, particularly for water-constrained systems and climates, even when capital investment is low.

Vertical flow constructed wetlands subject to hydrocarbon contamination are associated with an encouraging treatment performance. However, the corresponding yields are rather low. Filters associated with a high loading rate release more nutrients into their effluents, which results in a greater marketable profit. This applies to both uncontaminated vertical flow constructed wetlands and those with hydrocarbon contamination. Marketable yields were substantially higher for filters lacking hydrocarbon pollution. A subset of these wetlands, containing small aggregates and where the contact time and loading rate were low, provided good yields. In comparison, in wetlands subject to diesel spills, high yields of chillies in terms of economic return were linked to small aggregate size, high contact time, high loading rate and irrigation water based on concentrated wastewater. Some findings presented in this article show a good agreement with what has recently been published in the literature.

Regarding food contamination by poisonous elements, only slight zinc contamination was detected in harvested chillies for filter 8 (characterised predominantly by a low wetland resting time) based on European standards for vegetables. Furthermore, considering that the economic return for chillies irrigated with diesel-contaminated irrigation water is usually rather low, the authors recommend not releasing the corresponding harvest to the market. Further research on chilli fruit contamination by recycled treated domestic wastewater from constructed wetland systems should be studied at a field scale to assess the impact of accumulated contaminants on the growth of chilli fruits and their productivity in terms of yield and economic return. Considerable more research on optimising system performance for other plants receiving recycled pretreated wastewater from other treatment technologies should also be performed.

## Electronic supplementary material

ESM 1 (PDF 282 kb)

ESM 2 (PDF 30 kb)

ESM 3 (PDF 110 kb)

ESM 4 (PDF 26 kb)

ESM 5 (PDF 75 kb)

ESM 6 (PDF 94 kb)

ESM 7 (PDF 35 kb)
