# Supplementary material for: Assessment of diesel-contaminated domestic wastewater treated by constructed wetlands for irrigation of chillies grown in a greenhouse
Source: Environ Sci Pollut Res Int. 2016 Sep 27;23(24):25003–23. doi: 10.1007/s11356-016-7706-x (PMC5124056; doi:10.1007/s11356-016-7706-x)
Supplement: Supplementary file 1 — (PDF 282 kb) [file 11356_2016_7706_MOESM1_ESM.pdf]

Assessment of Diesel-Spilled Domestic Wastewater Treated by Vertical-Flow Constructed Wetlands for Irrigation of Chillies Grown in a Greenhouse

Environmental Science and Pollution Control

Rawaa H.K. Al-Isawi, Miklas Scholz* and Furat A. M. Al-Faraj

Civil Engineering Research Group, School of Computing, Science and Engineering, The University of Salford, Newton Building, Salford M5 4WT, England, United Kingdom

***e-mail:m.scholz@salford.ac.uk; Tel.: 0044-161-2955921; fax: 0044-161-2955575**

Online Resource 1 Comparison of the water quality of the inflow waters received by the Chilli pots (value, sample number (in brackets) and standard deviation, if applicable)

Parameter	Unit	Overall[a]	RPBF[b]	RPAF[c]	RPAFD[d]
Filter 1 (outflow)					
Total Petroleum Hydrocarbons	µg/l	1986(6)±1829.72	100(1)±nm	332(1)±nm	2872(4)±1560.5
Chemical oxygen demand	mg/l	133.8(22)±66.50	61.3(2)±3.04	78.9(9)±13.29	191.9(11)±40.79
Biochemical oxygen demand	mg/l	51.5(40)±30.20	38.0(4)±30.24	28.0(20)±11.09	84.1(16)±10.57
Ammonia-nitrogen	mg/l	4.5(21)±2.73	1.4(2)±0.39	5.2(10)±2.53	4.5(9)±2.89
Nitrate-nitrogen	mg/l	0.3(22)±0.10	0.3(2)±0.17	0.3(10)±0.13	0.3(10)±0.05
Ortho-phosphate-phosphorus	mg/l	6.0(23)±2.51	2.4(2)±0.13	5.4(10)±2.56	7.3(11)±1.78
Suspended solids	mg/l	17.3(41)±14.09	6.0(6)±5.55	9.7(21)±7.53	33.6(14)±8.49
Turbidity	NTU[e]	15.9(39)±13.93	6.1(6)±2.38	7.4(19)±3.94	31.5(14)±11.55
pH	–	6.2(40)±0.32	6.4(6)±0.15	6.2(20)±0.24	6.0(14)±0.38
Redox potential	mV	26.2(43)±6.22	18.2(5)±2.95	25.4(22)±4.99	29.8(16)±5.91
Conductivity	µS/cm	503.2(44)±208.83	318.1(5)±51.25	369.6(21)±22.23	710.4(18)±177.46
Dissolved oxygen	mg/l	1.5(40)±0.78	1.1(2)±0.57	1.7(21)±0.88	1.3(17)±0.64
Filter 2 (outflow)					
Total Petroleum Hydrocarbons	µg/l	<10	nm	<10	<10

Online Resource 1 (cont.)

Chemical oxygen demand	mg/l	35.5(18)±13.24	17.3(2)±1.63	32.2(9)±11.48	44.8(7)±9.59
Biochemical oxygen demand	mg/l	15.6(40)±8.50	16.0(4)±6.93	14.1(20)±8.17	17.3(16)±9.38
Ammonia-nitrogen	mg/l	5.2(21)±5.40	3.1(2)±1.21	6.5(10)±6.39	7.0(9)±4.64
Nitrate-nitrogen	mg/l	1(19)±1.80	7.2(2)±0.20	0.9(10)±1.33	0.4(8)±0.28
Ortho-phosphate-phosphorus	mg/l	3.8(20)±1.23	2.0(2)±0.15	3.8(10)±1.34	4.3(8)±0.77
Suspended solids	mg/l	7.9(41)±7.51	2.3(6)±2.25	6.3(21)±4.72	12.7(14)±9.74
Turbidity	NTU[e]	6.5(38)±6.27	2.8(6)±0.70	4.0(18)±1.48	11.2(14)±8.37
pH	–	6.5(40)±0.18	6.6(6)±0.12	6.5(20)±0.18	6.5(14)±0.20
Redox potential	mV	10.1(43)±6.16	6.6(5)±3.36	11.5(22)±5.01	9.3(16)±7.78
Conductivity	µS/cm	491.7(44)±171.61	307.3(5)±46.71	372.0(21)±24.84	682.6(18)±83.58
Dissolved oxygen	mg/l	2.0(40)±0.85	1.4(2)±0.49	2.1(21)±0.84	1.9(17)±0.87
Filter 3 (outflow)					
Total Petroleum Hydrocarbons	µg/l	1554(6)±1340.93	69(1)±nm	37(1)±nm	2305(4)±861.8
Chemical oxygen demand	mg/l	153.8(22)±86	73.2(2)±3.61	88.2(9)±20.28	221.7(11)±71.77
Biochemical oxygen demand	mg/l	37.4(41)±30.68	17.7(6)±9.42	18.5(19)±6.67	67.3(16)±29.44
Ammonia-nitrogen	mg/l	3.1(21)±2.24	0.8(2)±0.21	4.4(10)±2.51	2.3(9)±1.16
Nitrate-nitrogen	mg/l	0.3(22)±0.09	0.3(2)±0.20	0.3(10)±0.08	0.3(10)±0.07
Ortho-phosphate-phosphorus	mg/l	5.1(23)±2.38	1.9(2)±0.88	4.3(10)±2.18	6.4(11)±1.89
Suspended solids	mg/l	16.3(41)±13.65	5.5(6)±4.23	10.0(21)±9.45	30.4(14)±9.80
Turbidity	NTU[e]	13.4(38)±12.73	6.0(6)±2.25	4.9(18)±1.95	27.6(14)±10.53
pH	–	6.4(40)±0.21	6.6(6)±0.13	6.5(20)±0.15	6.2(14)±0.20
Redox potential	mV	16.3(43)±6.91	7.4(5)±4.04	13.1(22)±3.85	23.5(16)±3.50
Conductivity	µS/cm	593.3(44)±216.48	396.2(5)±109.90	439.9(21)±26.76	827.1(18)±128.37
Dissolved oxygen	mg/l	1.7(40)±0.96	2.0(2)±1.91	2.0(21)±0.95	1.3(17)±0.73
Filter 4 (outflow)					
Total Petroleum Hydrocarbons	µg/l	<10	nm	<10	<10
Chemical oxygen demand	mg/l	37.5(18)±19.03	10.9(2)±0.64	35.9(9)±21.30	47.2(7)±8.45
Biochemical oxygen demand	mg/l	14.4(41)±9.27	11.2(5)±8.67	16.3(20)±9.89	13.0(16)±8.64
Ammonia-nitrogen	mg/l	2.8(21)±2.81	0.1(2)±0.11	1.8(10)±2.14	4.1(9)±3.07
Nitrate-nitrogen	mg/l	0.5(22)±1.30	5.8(2)±1.87	0.1(10)±0.05	0.2(8)±0.16
Ortho-phosphate-phosphorus	mg/l	3.6(20)±1.24	1.8(2)±0.19	3.0(10)±0.93	4.7(8)±0.58
Suspended solids	mg/l	6.0(41)±4.95	6.3(6)±5.20	6.0(21)±5.18	5.7(14)±4.86
Turbidity	NTU[e]	4.3(38)±2.64	7.3(6)±3.10	4.0(18)±2.82	3.5(14)±0.94

Online Resource 1 (cont.)

pH	–	6.5(40)±0.19	6.5(6)±0.12	6.5(20)±0.23	6.5(14)±0.18
Redox potential	mV	12.1(43)±4.33	9.0(5)±2.74	12.2(22)±3.46	13.0(16)±5.44
Conductivity	µS/cm	500.4(44)±209.31	317.9(5)±67.12	398.5(21)±37.61	670.1(18)±234.52
Dissolved oxygen	mg/l	2.1(40)±0.99	1.3(2)±0.85	2.2(21)±0.90	2.2(17)±1.12
Filter 5 (outflow)					
Total Petroleum Hydrocarbons	µg/l	2698(6)±2016.75	14(1)±nm	218(1)±nm	3989(4)±324.2
Chemical oxygen demand	mg/l	149.8(22)±92.10	60.3(2)±0.35	73.9(9)±9.95	228.2(11)±64
Biochemical oxygen demand	mg/l	38.7(42)±29.70	9.0(6)±8.37	21.5(20)±9.86	71.3(16)±19.68
Ammonia-nitrogen	mg/l	10.1(22)±3.99	12.6(2)±2.05	11.0(10)±2.53	8.9(10)±5.15
Nitrate-nitrogen	mg/l	0.7(22)±0.77	1.5(2)±1.87	0.8(10)±0.89	0.5(10)±0.05
Ortho-phosphate-phosphorus	mg/l	7.2(23)±3.77	1.8(2)±0.89	5.3(10)±1.60	10.0(11)±3.25
Suspended solids	mg/l	15.8(41)±15.33	6.0(6)±4.34	7.2(21)±5.21	32.9(14)±13.97
Turbidity	NTU[e]	11.9(38)±10.67	4.7(6)±0.87	5.8(18)±2.19	22.7(14)±10.81
pH	–	6.5(39)±0.16	6.6(6)±0.14	6.5(19)±0.10	6.4(14)±0.14
Redox potential	mV	9.9(43)±8.51	5.0(5)±8.12	8.8(22)±3.24	12.9(16)±12.27
Conductivity	µS/cm	918.3(44)±361.21	507.1(5)±187.64	672.7(21)±63.86	1319.1(18)±150.56
Dissolved oxygen	mg/l	1.5(40)±0.77	1.4(2)±1.13	1.9(21)±0.71	1.1(17)±0.63
Filter 6 (outflow)					
Total Petroleum Hydrocarbons	µg/l	<10	nm	<10	<10
Chemical oxygen demand	mg/l	43.1(18)±21.25	24.9(2)±13.29	34.9(9)±23.52	58.8(7)±3.67
Biochemical oxygen demand	mg/l	21.2(42)±17.64	9.3(6)±8.91	16.9(20)±12.05	31.0(16)±21.35
Ammonia-nitrogen	mg/l	9.2(22)±7.21	0.5(2)±0.11	10.2(10)±8.58	9.1(10)±5.69
Nitrate-nitrogen	mg/l	3.8(21)±3.88	0.2(2)±1.87	3.4(10)±3.85	4.7(9)±4.05
Ortho-phosphate-phosphorus	mg/l	5.3(21)±2.83	2.9(2)±0.32	6.0(10)±3.66	5.0(9)±1.70
Suspended solids	mg/l	5.6(41)±4.77	4.2(6)±2.32	5.9(21)±5.93	5.8(14)±3.57
Turbidity	NTU[e]	4.4(38)±2.55	3.5(6)±1.57	4.9(18)±3.26	4.2(14)±1.69
pH	–	6.8(40)±0.17	6.8(6)±0.14	6.7(20)±0.16	6.8(14)±0.20
Redox potential	mV	2.0(43)±4.92	4.6(5)±5.27	2.6(22)±3.05	0.4(16)±6.48
Conductivity	µS/cm	882.7(44)±355.35	423.4(5)±126.88	661.4(21)±54.94	1268.6(18)±178.53
Dissolved oxygen	mg/l	2.0(40)±0.03	1.2(2)±0.42	2.1(21)±1.12	2.1(17)±0.95
Filter 7 (outflow)					
Total Petroleum Hydrocarbons	µg/l	<10	nm	<10	<10
Chemical oxygen demand	mg/l	27.8(18)±11.35	14.1(2)±0.07	31.0(9)±14.01	27.5(7)±4.97

Online Resource 1 (cont.)

Biochemical oxygen demand	mg/l	10.2(46)±5.59	15.0(6)±5.76	9.8(23)±5.72	8.9(17)±4.70
Ammonia-nitrogen	mg/l	4.2(23)±5.37	10.6(2)±14.32	4.3(12)±4.81	2.6(9)±2.96
Nitrate-nitrogen	mg/l	3.3(19)±3.33	8.3(2)±0.32	1.3(10)±0.36	5.6(7)±3.42
Ortho-phosphate-phosphorus	mg/l	4.2(20)±1.99	1.9(2)±0.13	4.3(10)±2.62	4.6(8)±0.49
Suspended solids	mg/l	2.7(52)±4.08	2.7(7)±2.98	4.0(27)±5.06	0.6(18)±0.50
Turbidity	NTU[e]	3.4(49)±2.61	2.7(7)±0.69	4.3(24)±3.30	2.4(18)±1.43
pH	–	6.6(54)±0.19	6.5(7)±0.21	6.6(26)±0.16	6.6(21)±0.23
Redox potential	mV	4.9(54)±5.79	7.4(7)±4.50	7.3(27)±4.08	0.9(20)±6.07
Conductivity	µS/cm	511.6(51)±179.00	337.0(5)±100.39	399.3(26)±99.31	701.1(20)±78.35
Dissolved oxygen	mg/l	2.2(49)±1.24	1.4(2)±0.85	1.8(26)±0.74	2.8(21)±1.52
Filter 8 (outflow)					
Total Petroleum Hydrocarbons	µg/l	1463(2)±2069.0	nm	2926(1)±nm	<10
Chemical oxygen demand	mg/l	48.0(18)±37.12	64.3(2)±0.42	28.1(9)±9.08	68.8(7)±51.03
Biochemical oxygen demand	mg/l	13.4(53)±5.64	14.3(8)±6.36	13.9(24)±6.58	12.5(21)±4.14
Ammonia-nitrogen	mg/l	1.4(21)±1.59	0.7(2)±0.12	1.5(10)±1.40	1.4(9)±1.93
Nitrate-nitrogen	mg/l	2.9(16)±2.22	6.6(2)±1.90	1.5(8)±1.43	3.9(7)±1.94
Ortho-phosphate-phosphorus	mg/l	3.6(19)±1.60	1.9(2)±0.10	3.6(10)±2.11	4.0(8)±0.58
Suspended solids	mg/l	2.9(54)±4.38	6.0(9)±7.55	3.0(29)±3.81	1.1(16)±1.00
Turbidity	NTU[e]	3.3(51)±2.99	5.4(9)±4.82	3.2(26)±2.68	2.4(16)±1.38
pH	–	6.5(56)±0.17	6.5(9)±0.10	6.5(29)±0.18	6.4(18)±0.18
Redox potential	mV	11.6(57)±6.65	14.5(8)±3.78	13.5(30)±5.92	7.4(19)±6.85
Conductivity	µS/cm	532.8(60)±214.52	315.3(7)±62.82	394.5(29)±99.60	763.3(24)±104.97
Dissolved oxygen	mg/l	2.0(55)±0.87	1.9(2)±1.06	2.0(28)±0.93	2.1(25)±0.83
Control A (outflow)					
Total Petroleum Hydrocarbons	µg/l	3081(6)±2269.2	345(1)±nm	1270(1)±nm	4218(4)±1808.1
Chemical oxygen demand	mg/l	96.1(22)±44.54	11.5(2)±6.58	94.3(9)±40.19	113.0(11)±33.50
Biochemical oxygen demand	mg/l	13.4(39)±6.05	9.6(5)±7.13	12.9(20)±4.96	15.6(14)±6.66
Ammonia-nitrogen	mg/l	0.3(21)±0.50	1.0(2)±0.67	0.3(10)±0.61	0.1(9)±0.11
Nitrate-nitrogen	mg/l	0.2(22)±0.11	0.2(2)±0.03	0.2(10)±0.05	0.2(10)±0.16
Ortho-phosphate-phosphorus	mg/l	2.0(23)±0.37	2.1(2)±0.04	2.0(10)±0.53	1.9(11)±0.19
Suspended solids	mg/l	10.9(41)±8.17	2.8(6)±2.79	13.4(21)±9.59	10.5(14)±4.55
Turbidity	NTU[e]	7.6(38)±3.51	3.0(6)±0.90	7.8(18)±3.71	9.3(14)±1.90
pH	–	6.6(40)±0.16	6.6(6)±0.08	6.7(20)±0.16	6.6(14)±0.17

Online Resource 1 (cont.)

Redox potential	mV	2.9(43)±3.33	1.6(5)±4.16	2.4(22)±3.55	4.0(16)±2.56
Conductivity	µS/cm	205.3(44)±72.73	135.3(5)±23.88	177.7(21)±17.81	256.8(18)±87.63
Dissolved oxygen	mg/l	1.5(40)±0.78	1.2(2)±0.92	1.5(21)±0.77	1.6(17)±0.83
Control B (outflow)					
Total Petroleum Hydrocarbons	µg/l	<10	nm	<10	<10
Chemical oxygen demand	mg/l	9.9(18)±7.28	8.3(2)±4.14	12.2(9)±9.13	7.4()±
Biochemical oxygen demand	mg/l	8.4(40)±5.53	11.0(6)±7.77	9.4(20)±4.77	5.9(14)±4.87
Ammonia-nitrogen	mg/l	0.5(21)±1.59	6.9(2)±0.12	0.3(10)±0.72	0.0(9)±0.03
Nitrate-nitrogen	mg/l	0.1(19)±0.05	0.2(2)±0.12	0.1(10)±0.02	0.0(7)±0.03
Ortho-phosphate-phosphorus	mg/l	1.9(20)±0.32	2.2(2)±0.15	2.0(10)±0.20	1.6(8)±0.34
Suspended solids	mg/l	1.7(40)±2.19	1.5(6)±1.22	2.0(21)±2.70	1.3(13)±1.58
Turbidity	NTU[e]	2.6(38)±0.79	2.4(6)±0.16	2.5(18)±0.88	2.8(14)±0.83
pH	–	6.5(39)±0.18	6.5(6)±0.13	6.5(20)±0.17	6.6(13)±0.20
Redox potential	mV	10.8(42)±4.18	13.4(5)±3.44	12.8(22)±3.30	7.1(15)±2.76
Conductivity	µS/cm	176.7(43)±42.64	149.6(5)±26.40	175.9(21)±21.34	185.6(17)±60.96
Dissolved oxygen	mg/l	2.9(40)±1.38	1.4(2)±1.13	2.2(21)±0.94	3.9(17)±1.19
Deionised water					
Total Petroleum Hydrocarbons	µg/l	nm	nm	nm	nm
Chemical oxygen demand	mg/l	0.0(4) ±0	0.0(1)	0.0(1)	0.0(2)±0
Biochemical oxygen demand	mg/l	0.4(16)±0.63	0.0(4)±0	0.6(8)±0.74	0.5(4)±0.44
Ammonia-nitrogen	mg/l	0.0(2)±0	0.0(1)±nm	0.0(1)±nm	nm
Nitrate-nitrogen	mg/l	0.0(2)±0	0.0(1)±nm	0.0(1)±nm	nm
Ortho-phosphate-phosphorus	mg/l	0.0(2)±0	0.0(1)±nm	nm	0.0(1)±nm
Suspended solids	mg/l	0.5(25)±0.59	0.3(6)±0.82	0.7(12)±0.43	0.4(7)±0.76
Turbidity	NTU[e]	0.3(25)±0.40	0.2(5)±0.34	0.3(13)±0.37	0.5(7)±0.51
pH	–	6.0(24)±0.76	6.1(7)±1.08	5.8(10)±0.69	6.3(7)±0.29
Redox potential	mV	27.3(11)±34.08	57.2(3)±54.48	17.7(6)±19.69	11.5(2)±2.12
Conductivity	µS/cm	4.1(11)±2.44	3.5(4)±3.17	6.1(4)±0.06	2.2(3)±0.97
Dissolved oxygen	mg/l	8.0(9)±0.94	8.3(1)±nm	7.9(4)±0.85	7.9(4)±0.85
Tap water (100%)					
Total Petroleum Hydrocarbons	µg/l	nm	nm	nm	nm
Chemical oxygen demand	mg/l	2.3(6)±0.08	2.3(2)±0.07	2.3(2)±0.14	2.3(2)±0.07
Biochemical oxygen demand	mg/l	2.6(18)±1.37	5.0(2)±1.41	2.3(10)±0.82	2.5(6)±1.52

Online Resource 1 (cont.)

Ammonia-nitrogen	mg/l	0.2(8)±0.06	0.1(2)±0.05	0.2(2)±0.07	0.2(4)±0.09
Nitrate-nitrogen	mg/l	0.2(9)±0.10	0.4(2)±0.08	0.1(3)±nm	0.2(4)±0.17
Ortho-phosphate-phosphorus	mg/l	0.6(7)±0.20	0.5(3)±0.05	nm	0.7(4)±0.30
Suspended solids	mg/l	0.9(25)±1.09	0.7(6)±1.63	0.8(12)±0.97	1.3(7)±0.76
Turbidity	NTU[e]	1.3(25)±1.09	1.1(5)±0.79	2.0(13)±0.73	0.8(7)±0.42
pH	–	6.3(24)±0.70	5.9(7)±0.96	6.4(10)±0.66	6.4(7)±0.32
Redox potential	mV	23.9(15)±12.12	27.7(3)±6.47	23.9(10)±14.30	18.0(2)±4.24
Conductivity	µS/cm	77.2(11)±9.59	75.2(4)±10.61	85.0(4)±5.77	69.4(3)±4.61
Dissolved oxygen	mg/l	8.9(9)±0.53	9.4(1)±nm	9.1(4)±0.27	8.5(4)±0.58
Tap water with fertiliser (0.7 ml/l)					
Total Petroleum Hydrocarbons	µg/l	nm	nm	nm	nm
Chemical oxygen demand	mg/l	2.5(6)±0.10	2.5(2)±0	2.6(2)±0.07	2.4(2)±0.14
Biochemical oxygen demand	mg/l	15.1(17)±11.67	8.7(3)±1.53	17.6(10)±13.36	13.5(4)±11.12
Ammonia-nitrogen	mg/l	2.4(7)±0.07	2.4(2)±0.07	2.5(2)±0.07	2.4(3)±0.06
Nitrate-nitrogen	mg/l	5.6(3)±0.13	5.6(1)±nm	5.6(2)±0.18	nm
Ortho-phosphate-phosphorus	mg/l	4.4(2)±0.07	4.3(1)±nm	nm	4.4(1)±nm
Suspended solids	mg/l	2.2(25)±1.29	1.3(6)±0.82	2.3(12)±1.56	2.7(7)±0.76
Turbidity	NTU[e]	3.1(25)±1.53	3.0(5)±0.56	3.7(13)±1.54	2.3(7)±1.73
pH	–	6.3(23)±0.25	6.2(6)±0.20	6.2(10)±0.22	6.5(7)±0.25
Redox potential	mV	27.6(9)±18.79	56.1(1)±nm	24.5(6)±18.93	22.5(2)±10.61
Conductivity	µS/cm	185.9(7)±67.88	211.0(2)±57.98	175.0(4)±86.60	179.0(1)±nm
Dissolved oxygen	mg/l	7.2(9)±0.99	8.1(1)±nm	7.3(4)±0.98	7.0(4)±1.15
Wastewater (20%); tap water (80%)					
Total Petroleum Hydrocarbons	µg/l	nm	nm	nm	nm
Chemical oxygen demand	mg/l	48.4(22)±19.98	41.2(2)±26.59	48.6(12)±14.62	49.91(12)±27.25
Biochemical oxygen demand	mg/l	28.4(42)±20.36	12.7(3)±11.02	17.5(22)±14.25	45.4(17)±16.26
Ammonia-nitrogen	mg/l	8.0(23)±2.96	4.1(2)±4.92	7.5(12)±2.78	9.7(9)±1.77
Nitrate-nitrogen	mg/l	0.2(23)±0.19	0.5(2)±0.49	0.3(12)±0.16	0.1(9)±0.07
Ortho-phosphate-phosphorus	mg/l	3.0(24)±0.87	2.6(2)±0.14	3.4(12)±1.11	2.68(10)±0.25
Suspended solids	mg/l	49.0(25)±23.44	26.0(6)±26.89	55.1(12)±12.30	58.1(7)±24.84
Turbidity	NTU[e]	18.5(25)±11.83	9.1(5)±3.55	17.7(13)±12.96	26.8(7)±7.68
pH	–	7.1(23)±0.28	7.0(6)±0.22	7.1(10)±0.22	7.1(7)±0.43
Redox potential	mV	-4.7(39)±10.41	-7.2(7)±3.35	-3.8(22)±11.08	-4.8(10)±12.53

Online Resource 1 (cont.)

Conductivity	µS/cm	182.1(47)±105.19	84.4(7)±51.65	139.6(24)±15.43	288.6(16)±114.26
Dissolved oxygen	mg/l	8.2(41)±2.63	3.8(2)±3.18	7.3(23)±2.49	10.0(16)±1.03
Wastewater (100%)					
Total Petroleum Hydrocarbons	µg/l	271(2)±318.20	nm	496(1)	46(1)
Chemical oxygen demand	mg/l	257.0(22)±85.56	206.0(2)±132.94	249.7(13)±74.04	285.2(7)±99.02
Biochemical oxygen demand	mg/l	124.9(43)±84.58	48.0(5)±32.71	84.3(21)±60.05	197.7(17)±66.10
Ammonia-nitrogen	mg/l	40.2(23)±14.80	20.5(2)±24.61	37.3(12)±13.88	48.3(9)±8.84
Nitrate-nitrogen	mg/l	1.1(23)±0.93	2.4(2)±2.43	1.2(12)±0.80	0.6(9)±0.16
Ortho-phosphate-phosphorus	mg/l	13.4(26)±5.54	12.1(2)±1.98	14.1(13)±7.85	12.8(11)±0.93
Suspended solids	mg/l	146.2(48)±76.17	145.7(6)±74.06	136.0(26)±82.68	162.8(16)±66.98
Turbidity	NTU[e]	70.7(43)±47.52	63.3(6)±45.59	81.0(21)±60.12	59.9(16)±22.76
pH	–	7.7(44)±0.48	7.4(6)±0.22	7.6(22)±0.55	8.0(16)±0.21
Redox potential	mV	-42.4(43)±18.39	-36.0(7)±16.75	-38.3(21)±19.48	-51.1(15)±15.05
Conductivity	µS/cm	965.4(52)±503.52	448.6(6)±110.66	689.9(28)±79.81	1566.7(18)±380.48
Dissolved oxygen	mg/l	7.4(43)±2.81	3.5(2)±3.61	6.5(23)±2.99	9.0(18)±1.22

[a]Overall period: 08/04/14 to 24/12/14;

[b]Replanting period before fruiting: 08/04/14 to 11/05/14;

[c]Replanting period after fruiting: 12/05/14 to 25/09/14;

[c]Replanting period after fruiting (second diesel spill on 26/09/14): 26/09/14 to 24/12/14;

[e]nephelometric turbidity unit; and

Note: nm, not measured
