# Supplementary material for: Assessment of diesel-contaminated domestic wastewater treated by constructed wetlands for irrigation of chillies grown in a greenhouse
Source: Environ Sci Pollut Res Int. 2016 Sep 27;23(24):25003–23. doi: 10.1007/s11356-016-7706-x (PMC5124056; doi:10.1007/s11356-016-7706-x)
Supplement: Supplementary file 2 — (PDF 30 kb) [file 11356_2016_7706_MOESM2_ESM.pdf]

Assessment of Diesel-Spilled Domestic Wastewater Treated by Vertical-Flow Constructed Wetlands for Irrigation of Chillies Grown in a Greenhouse

Environmental Science and Pollution Control

Rawaa H.K. Al-Isawi, Miklas Scholz* and Furat A. M. Al-Faraj

R.H.K. Al-Isawi • M. Scholz • F.A.M Al-Faraj
Civil Engineering Research Group, School of Computing, Science and Engineering, The University of Salford, Newton Building, Salford M5 4WT, England, United Kingdom

***e-mail:m.scholz@salford.ac.uk; Tel.: 0044-161-2955921; fax: 0044-161-2955575**

Online Resource 2 Overview of the statistically significant differences (indicated by *p*-value (*h*)) between outflow water quality variables of different wetland systems using the non-parametric Mann-Whitney U-test for data collected between 8 April 2014 and 24 December 2014

Parameter	COD[a]	NH_4-N[b]	NO_3-N[c]	PO_4-P[d]	Redox[e]	EC[f]
Unit	mg/l	mg/l	mg/l	mg/l	mv	µS/cm
Potential differences between the diesel-contaminated wetland filters (set 1)						
Wetlands 1 and 3[g]	0.331 (0)	0.131 (0)	0.970 (0)	0.163 (0)	0.000 (1)	0.004 (1)
Wetlands 3 and 5[h]	0.490 (0)	0.000 (1)	0.002 (1)	0.044 (1)	0.000 (1)	0.010 (1)
Potential differences between wetland filters without diesel contamination (set 2)						
Wetlands 2 and 4[g]	0.650 (0)	0.006 (1)	0.012 (1)	0.547 (0)	0.000 (1)	0.491 (0)
Wetlands 4 and 6[h]	0.293 (0)	0.001 (1)	0.000 (1)	0.102 (0)	0.000 (1)	0.000 (1)
Wetlands 4 and 7[i]	0.226 (0)	0.488 (0)	0.000 (1)	0.495 (0)	0.000 (1)	0.556 (0)
Wetlands 7 and 8[j]	0.029 (1)	0.144 (0)	0.757 (0)	0.296 (0)	0.000 (1)	0.710 (0)

Note: *p*-value, probability of obtaining a test statistic at least as extreme as the one that was actually observed, assuming that the null hypothesis is true; *h*, response indicator (shown in brackets); if *h*=1, units are statistically significantly different (*p*-value<0.05) for the corresponding water quality parameter; if *h*=0, the difference is not statistically significantly different (*p*-value>0.05).
[a]chemical oxygen demand;
[b]ammonia-nitrogen;
[c]nitrate-nitrogen;
[d]ortho-phosphate-phosphorus;
[e]redox potential;
[f]electric conductivity;
[g]influence of aggregate diameter (Table 1);
[h]influence of inflow COD load (Table 1);
[i]influence of contact time (Table 1); and
[j]influence of resting time (Table 1).
