# Supplementary material for: Assessment of diesel-contaminated domestic wastewater treated by constructed wetlands for irrigation of chillies grown in a greenhouse
Source: Environ Sci Pollut Res Int. 2016 Sep 27;23(24):25003–23. doi: 10.1007/s11356-016-7706-x (PMC5124056; doi:10.1007/s11356-016-7706-x)
Supplement: Supplementary file 3 — (PDF 110 kb) [file 11356_2016_7706_MOESM3_ESM.pdf]

Assessment of Diesel-Spilled Domestic Wastewater Treated by Vertical-Flow Constructed Wetlands for Irrigation of Chillies Grown in a Greenhouse

Environmental Science and Pollution Control

Rawaa H.K. Al-Isawi, Miklas Scholz* and Furat A. M. Al-Faraj

R.H.K. Al-Isawi • M. Scholz • F.A.M Al-Faraj
Civil Engineering Research Group, School of Computing, Science and Engineering, The University of Salford, Newton Building, Salford M5 4WT, England, United Kingdom

***e-mail:m.scholz@salford.ac.uk; Tel.: 0044-161-2955921; fax: 0044-161-2955575**

Online Resource 3 Overview of total water volumes for Chilli (C) plants for different planting periods

Plant	Total irrigation water volume (l)				Plant	Total irrigation water volume (l)			
	FRP[a]	SRPBF[b]	SRPAF[c]	SRPAFD[d]		FRP[a]	SRPBF[b]	SRPAF[c]	SRPAFD[d]
C1	0.25	2.40	14.83	5.90	C46	0.23	2.95	17.80	7.20
C2	0.24	1.95	14.98	5.45	C47	0.23	2.95	17.35	6.80
C3	0.23	2.35	15.08	5.30	C48	0.24	2.75	16.55	6.80
C4	0.25	2.70	14.53	5.35	C49	0.25	2.70	15.03	5.90
C5	0.26	2.20	14.38	5.75	C50	0.23	2.75	15.48	6.00
C6	0.24	2.50	14.63	5.65	C51	0.25	2.20	14.68	5.60
C7	0.25	2.70	17.90	6.80	C52	0.25	2.40	15.38	5.85
C8	0.26	2.50	18.08	6.70	C53	0.25	1.65	14.70	5.75
C9	0.24	2.25	17.00	6.55	C54	0.25	2.50	15.00	5.70
C10	0.25	2.55	17.35	6.55	C55	0.25	2.40	17.00	6.25
C11	0.26	2.60	16.85	6.25	C56	0.24	2.40	17.10	6.25
C12	0.24	2.70	16.85	6.05	C57	0.24	2.05	16.45	6.25
C13	0.23	2.80	16.75	5.93	C58	0.26	2.50	15.90	6.15
C14	0.27	2.35	16.50	5.60	C59	0.26	2.10	16.15	6.30
C15	0.25	1.70	16.30	5.00	C60	0.27	1.85	14.65	6.20
C16	0.25	2.45	16.30	5.25	C61	0.30	2.55	15.90	5.75
C17	0.25	2.40	15.70	5.80	C62	0.30	2.55	16.25	5.55
C18	0.24	2.70	16.40	5.50	C63	0.30	2.55	15.90	5.55
C19	0.24	2.45	17.90	6.48	C64	0.31	2.55	15.75	5.45
C20	0.24	2.45	17.50	6.38	C65	0.32	2.80	16.30	5.45
C21	0.24	2.45	17.40	6.15	C66	0.31	2.60	16.60	5.55
C22	0.24	2.40	17.40	6.30	C67	0.31	2.45	16.55	6.10
C23	0.25	2.45	17.40	6.10	C68	0.29	2.55	16.65	6.65
C24	0.23	2.50	17.05	6.25	C69	0.28	2.55	17.40	6.75
C25	0.27	2.80	17.30	5.85	C70	0.32	2.55	17.40	6.20
C26	0.23	2.75	17.30	6.50	C71	0.32	2.75	17.45	6.15
C27	0.25	2.85	17.15	6.15	C72	0.31	2.75	17.25	6.45
C28	0.24	2.55	17.20	6.40	C73	0.29	3.00	18.15	6.50
C29	0.25	2.45	17.95	6.50	C74	0.28	3.00	18.05	6.55
C30	0.24	2.65	18.05	6.60	C75	0.29	3.00	17.50	6.55
C31	0.24	3.05	19.20	7.25	C76	0.29	3.00	17.60	6.65
C32	0.25	2.75	17.75	7.15	C77	0.31	3.10	17.75	6.80
C33	0.26	2.70	18.50	6.40	C78	0.31	3.00	16.55	6.65
C34	0.26	3.05	18.20	6.80	C79	0.32	3.05	17.58	7.50
C35	0.26	2.40	17.70	6.20	C80	0.33	3.05	17.58	7.60
C36	0.27	2.55	16.75	5.55	C81	0.30	2.55	17.68	7.75

Online Resource 3 (cont.)

C37	0.26	2.85	18.00	6.28	C82	0.32	2.85	18.23	8.10
C38	0.27	2.80	18.35	6.80	C83	0.31	2.80	18.43	7.85
C39	0.27	2.75	18.25	7.15	C84	0.32	2.45	17.48	7.60
C40	0.24	2.20	18.00	6.60	C85	0.34	2.75	18.40	7.50
C41	0.25	2.75	18.15	6.25	C86	0.32	3.00	17.75	7.20
C42	0.25	2.45	17.50	6.48	C87	0.30	2.80	17.75	7.20
C43	0.25	2.25	17.50	6.65	C88	0.31	2.75	18.05	7.35
C44	0.26	2.40	15.95	6.60	C89	0.29	2.75	17.70	7.05
C45	0.26	2.95	17.80	7.10	C90	0.28	2.75	17.80	7.05

[a]First replanting period: 12/02/14 to 07/04/14;
[b]Second replanting period before fruiting: 08/04/14 to 11/05/14;
[c]Second replanting period after fruiting:12/05/14 to 25/09/14; and
[d]Second replanting period after fruiting (second diesel spill on 26/09/14): 26/09/14 to 24/12/14
