# Supplementary material for: Assessment of diesel-contaminated domestic wastewater treated by constructed wetlands for irrigation of chillies grown in a greenhouse
Source: Environ Sci Pollut Res Int. 2016 Sep 27;23(24):25003–23. doi: 10.1007/s11356-016-7706-x (PMC5124056; doi:10.1007/s11356-016-7706-x)
Supplement: Supplementary file 4 — (PDF 26 kb) [file 11356_2016_7706_MOESM4_ESM.pdf]

**Assessment of Diesel-Spilled Domestic Wastewater Treated by Vertical-Flow Constructed Wetlands for Irrigation of Chillies Grown in a Greenhouse**

**Environmental Science and Pollution Control**

**Rawaa H.K. Al-Isawi, Miklas Scholz* and Furat A. M. Al-Faraj**

Civil Engineering Research Group, School of Computing, Science and Engineering, The University of Salford, Newton Building, Salford M5 4WT, England, United Kingdom

***e-mail:m.scholz@salford.ac.uk; Tel.: 0044-161-2955921; fax: 0044-161-2955575

**Online Resource 4** Overview of total number of buds, flowers and fruits for chilli (C) plants until 24 December 2014

| Inflow source | Total bud number | Total flower number | Total fruit number before harvest | Total fruit number after harvest |
|---|---|---|---|---|
| Filter 1 | C1(91);C2(83);C3(87);<br>C4(58);C5(70);C6(25) | C1(69);C2(50);C3(67);<br>C4(50);C5(57);C6(20) | C1(53);C2(33);C3(36);<br>C4(39);C5(41);C6(15) | C1(46);C2(30);C3(31);<br>C4(34);C5(40);C6(15) |
| Filter 2 | C7(83);C8(99);C9(79);<br>C10(80);C11(87);C12(39) | C7(74);C8(64);C9(67);<br>C10(66);C11(49);C12(33) | C7(72);C8(73);C9(56);<br>C10(46);C11(55);C12(18) | C7(70);C8(71);C9(53);<br>C10(43);C11(49);C12(18) |
| Filter 3 | C13(86);C14(50);C15(83);<br>C16(77);C17(55);C18(26) | C13(79);C14(42);C15(71);<br>C16(51);C17(44);C18(20) | C13(38);C14(36);C15(47);<br>C16(36);C17(24);C18(23) | C13(34);C14(33);C15(42);<br>C16(35);C17(21);C18(23) |
| Filter 4 | C19(90);C20(83);C21(56);<br>C22(97);C23(75);C24(53) | C19(70);C20(54);C21(47);<br>C22(53);C23(39);C24(49) | C19(54);C20(64);C21(40);<br>C22(40);C23(31);C24(37) | C19(52);C20(60);C21(40);<br>C22(39);C23(29);C24(36) |
| Filter 5 | C25(63);C26(82);C27(61);<br>C28(60);C29(55);C30(34) | C25(54);C26(50);C27(43);<br>C28(54);C29(47);C30(25) | C25(17);C26(21);C27(30);<br>C28(19);C29(20);C30(17) | C25(16);C26(19);C27(29);<br>C28(17);C29(19);C30(15) |
| Filter 6 | C31(72);C32(88);C33(73);<br>C34(74);C35(54);C36(25) | C31(40);C32(59);C33(57);<br>C34(66);C35(47);C36(20) | C31(39);C32(39);C33(32);<br>C34(44);C35(32);C36(15) | C31(34);C32(39);C33(30);<br>C34(43);C35(30);C36(15) |
| Filter 7 | C37(78);C38(113);C39(83);<br>C40(91);C41(79);C42(33) | C37(68);C38(65);C39(69);<br>C40(61);C41(61);C42(26) | C37(66);C38(63);C39(72);<br>C40(58);C41(46);C42(32) | C37(60);C38(61);C39(70);<br>C40(54);C41(44);C42(29) |
| Filter 8 | C43(115);C44(122);C45(98);<br>C46(111);C47(103);C48(64) | C43(109);C44(86);C45(90);<br>C46(98);C47(61);C48(43) | C43(83);C44(58);C45(80);<br>C46(86);C47(57);C48(33) | C43(79);C44(57);C45(78);<br>C46(84);C47(56);C48(31) |

Online Resource 4 (cont.)

| | | | | |
|---|---|---|---|---|
| Control A | C49(117);C50(79);C51(111); C52(102);C53(91);C54(53) | C49(98);C50(69);C51(75); C52(89);C53(82);C54(29) | C49(92);C50(55);C51(70); C52(73);C53(61);C54(18) | C49(91);C50(53);C51(69); C52(71);C53(60);C54(16) |
| Control B | C55(86);C56(112);C57(104); C58(110);C59(64);C60(25) | C55(71);C56(94);C57(84); C58(71);C59(58);C60(19) | C55(48);C56(84);C57(51); C58(62);C59(45);C60(14) | C55(48);C56(82);C57(50); C58(61);C59(40);C60(14) |
| Deionised water | C61(74);C62(67);C63(68); C64(58);C65(69);C66(54) | C61(58);C62(52);C63(59); C64(49);C65(62);C66(41) | C61(33);C62(36);C63(30); C64(27);C65(32);C66(31) | C61(29);C62(32);C63(29); C64(25);C65(29);C66(27) |
| Tap water | C67(99);C68(88);C69(68); C70(89);C71(82);C72(86) | C67(76);C68(52);C69(46); C70(69);C71(60);C72(54) | C67(66);C68(47);C69(38); C70(40);C71(40);C72(41) | C67(60);C68(43);C69(34); C70(40);C71(39);C72(40) |
| Tap water/fertiliser | C73(92);C74(131);C75(134); C76(133);C77(84);C78(121) | C73(82);C74(121);C75(87); C76(73);C77(77);C78(112) | C73(70);C74(103);C75(66); C76(61);C77(49);C78(92) | C73(70);C74(102);C75(66); C76(59);C77(47);C78(90) |
| Wastewater/tap | C79(128);C80(120);C81(116); C82(122);C83(93);C84(114) | C79(96);C80(72);C81(105); C82(87);C83(74);C84(89) | C79(78);C80(56);C81(100); C82(66);C83(67);C84(84) | C79(75);C80(53);C81(98); C82(64);C83(65);C84(81) |
| Wastewater | C85(115);C86(130);C87(109); C88(91);C89(104);C90(97) | C85(105);C86(103);C87(62); C88(49);C89(81);C90(82) | C85(86);C86(74);C87(56); C88(43);C89(71);C90(77) | C85(82);C86(71);C87(54); C88(41);C89(70);C90(73) |
