# Supplementary material for: Assessment of diesel-contaminated domestic wastewater treated by constructed wetlands for irrigation of chillies grown in a greenhouse
Source: Environ Sci Pollut Res Int. 2016 Sep 27;23(24):25003–23. doi: 10.1007/s11356-016-7706-x (PMC5124056; doi:10.1007/s11356-016-7706-x)
Supplement: Supplementary file 5 — (PDF 75 kb) [file 11356_2016_7706_MOESM5_ESM.pdf]

Assessment of Diesel-Spilled Domestic Wastewater Treated by Vertical-Flow Constructed Wetlands for Irrigation of Chillies Grown in a Greenhouse

Environmental Science and Pollution Control

Rawaa H.K. Al-Isawi, Miklas Scholz* and Furat A. M. Al-Faraj

R.H.K. Al-Isawi • M. Scholz • F.A.M Al-Faraj
Civil Engineering Research Group, School of Computing, Science and Engineering, The University of Salford, Newton Building, Salford M5 4WT, England, United Kingdom

***e-mail:m.scholz@salford.ac.uk; Tel.: 0044-161-2955921; fax: 0044-161-2955575

Online Resource 5 Overview of the statistically significant differences between Chilli fruit variables of different wetland filters and five types of irrigated water using the non-parametric Mann-Whitney U-test (08/04/14-24/12/14)

Parameter	Unit	Statistic	Aggregate diameter[a]	Contact time[b]	Resting time[c]	Inflow chemical oxygen demand load[d]
For filters without hydrocarbon						
Weight	g	*P-value*	0.657	0.005	<0.000	0.220
		h	0	1	1	0
Length	mm	*P-value*	0.206	<0.000	<0.000	<0.000
		h	0	1	1	1
Width	mm	*P-value*	0.605	<0.000	<0.000	0.004
		h	0	1	1	1
Bending	-	*P-value*	0.268	0.001	<0.000	0.311
		h	0	1	1	0
No. Buds	-	*P-value*	0.810	0.200	0.749	0.025
		h	0	0	0	1
No. Flowers	-	*P-value*	0.337	0.109	0.251	0.037
		h	0	0	0	1
No. Fruits	-	*P-value*	0.199	0.109	0.262	0.078
		h	0	0	0	0
Total price	£	*P-value*	0.902	0.773	0.688	0.695
		h	0	0	0	0
Parameter	Unit	Statistics	Aggregate diameter[e]	Contact time[f]	Resting time[g]	Inflow chemical oxygen demand load[h]
For filters with hydrocarbon						
Weight	g	*P-value*	<0.000	<0.000	<0.000	<0.000
		h	1	1	1	1
Length	mm	*P-value*	<0.000	<0.000	<0.000	<0.000
		h	1	1	1	1
Width	mm	*P-value*	0.070	<0.000	<0.000	<0.000
		h	0	1	1	1
Bending	-	*P-value*	<0.000	<0.000	<0.000	<0.000
		h	1	1	1	1
No. Buds	-	*P-value*	0.470	0.078	0.749	0.229
		h	0	0	0	0
No. Flowers	-	*P-value*	1.000	0.109	0.251	0.521
		h	0	0	0	0
No. Fruits	-	*P-value*	0.629	0.045	0.262	0.037
		h	0	0	0	1

Online Resource 5 (cont.)

Total price	£	*P-value*	0.312	0.523	0.688	0.031
		h	0	0	0	1
Parameter	Unit	Statistics	Filters 1 and 2	Filters 3 and 4	Filters 5 and 6	Control A and B
Comparison between filters with and without hydrocarbon						
Weight	g	*P-value*	<0.000	<0.000	<0.000	<0.001
		h	1	1	1	1
Length	mm	*P-value*	<0.000	<0.000	<0.000	<0.000
		h	1	1	1	1
Width	mm	*P-value*	<0.000	<0.000	<0.000	<0.000
		h	1	1	1	1
Bending	l	*P-value*	<0.000	0.037	<0.000	<0.000
		h	1	1	1	1
No. Buds	-	*P-value*	0.630	0.297	0.109	0.522
		h	0	0	0	0
No. Flowers	-	*P-value*	0.228	0.810	0.037	0.574
		h	0	0	1	0
No. Fruits	-	*P-value*	0.055	0.108	0.261	0.053
		h	0	0	0	0
Total price	£	*P-value*	0.097	0.108	0.053	0.062
		h	0	0	0	0
Parameter	Unit	Statistics	Lack of nutrients[i]	Fertiliser[j]	Diluted tab water[k]	Diluted waste-water[l]
Weight	g	*P-value*	<0.000	0.011	<0.000	<0.000
		h	1	1	1	1
Length	mm	*P-value*	<0.000	0.029	<0.000	<0.000
		h	1	1	1	1
Width	mm	*P-value*	<0.000	0.453	0.002	<0.000
		h	1	0	1	1
Bending	-	*P-value*	<0.000	<0.000	<0.000	<0.000
		h	1	1	1	1
No. Buds	-	*P-value*	0.013	0.037	0.150	0.262
		h	1	1	0	0
No. Flowers	-	*P-value*	0.378	0.006	0.010	0.810
		h	0	1	1	0
No. Fruits	-	*P-value*	0.004	0.013	0.008	0.575
		h	1	1	1	0
Total price	£	*P-value*	0.353	0.332	0.288	0.829
		h	0	0	0	0

[a]Comparison between the mean daily values of Filter 2, and the mean daily values of Filter 4;
[b]Comparison between the mean daily values of Filter 4, and Filter 7;
[c]Comparison between Filters 7 and 8;
[d]Comparison between mean daily values of Filter 4, and mean daily values of Filter 6;
[e]Comparison between the mean daily values of Filter 1, and the mean daily values of Filter 3;
[f]Comparison between the mean daily values of Filter 3, and Filter 7;
[g]Comparison between Filters 7 and 8;
[h]Comparison between mean daily values of Filter 3, and mean daily values of Filter 5;
[i]Comparison between the mean daily values of deionised water and the mean daily values of tap water;
[j]Comparison between the mean daily values of tap water (100%) and tap water with fertilizer (0.7ml/l);
[k]Comparison between tap water (100%) and tap water (80%) with wastewater (20%); and
[l]Comparison between mean daily values of tap water (80%) with wastewater (20%) and mean daily values of wastewater (100%).

Note: *P-value*, probability of obtaining a test statistic at least as extreme as the one that was actually observed, assuming that the null hypothesis is true; h, response indicator; if *h*=1, filters are statistically significantly different (*P-value*< 0.05) for the corresponding water quality parameter; if *h*=0, the difference is not significant
