# Supplementary material for: Assessment of diesel-contaminated domestic wastewater treated by constructed wetlands for irrigation of chillies grown in a greenhouse
Source: Environ Sci Pollut Res Int. 2016 Sep 27;23(24):25003–23. doi: 10.1007/s11356-016-7706-x (PMC5124056; doi:10.1007/s11356-016-7706-x)
Supplement: Supplementary file 6 — (PDF 94 kb) [file 11356_2016_7706_MOESM6_ESM.pdf]

Assessment of Diesel-Spilled Domestic Wastewater Treated by Vertical-Flow Constructed Wetlands for Irrigation of Chillies Grown in a Greenhouse

Environmental Science and Pollution Control

Rawaa H.K. Al-Isawi, Miklas Scholz* and Furat A. M. Al-Faraj

Civil Engineering Research Group, School of Computing, Science and Engineering, The University of Salford, Newton Building, Salford M5 4WT, England, United Kingdom

*****e-mail:m.scholz@salford.ac.uk; Tel.: 0044-161-2955921; fax: 0044-161-2955575

Online Resource 6 Chilli (C) harvest classification scheme (after Almuktar et al., 2015)

Variable	Class A	Class B	Class C	Class D	Class E
Quality class	Outstanding	Good	Good	Satisfactory	Unsatisfactory
Mean price Pence (Sterling)/gram	C: 2.00	C: 1.00	C: 0.50	C: 0.25	C: 0.00
Length (L, mm)	Very long (L≥80)	Long (60≤ L<80)	Medium (40≤ L<60)	Short (20≤L<40)	Very short (L<20)
Width (W, mm)	Very wide(W≥20)	Wide (16≤W<20)	Medium (12≤W<16)	Slim (8≤W<12)	Very slim (W<8)
Weight (w, g)	Very Large(w≥9)	Large (7≤w<9)	Medium (5≤w≤7)	Small (3≤w<5)	Very Small (w<3)
Bending	Characteristically bend; L/W≥3.5	Characteristically bend; L/W≥3.5	Characteristically bend; L/W≥3.5	Uncharacteristically bend; L/W<3.5	Uncharacteristically bend; L/W<3.5
