# Supplementary material for: Assessment of diesel-contaminated domestic wastewater treated by constructed wetlands for irrigation of chillies grown in a greenhouse
Source: Environ Sci Pollut Res Int. 2016 Sep 27;23(24):25003–23. doi: 10.1007/s11356-016-7706-x (PMC5124056; doi:10.1007/s11356-016-7706-x)
Supplement: Supplementary file 7 — (PDF 35 kb) [file 11356_2016_7706_MOESM7_ESM.pdf]

Assessment of Diesel-Spilled Domestic Wastewater Treated by Vertical-Flow Constructed Wetlands for Irrigation of Chillies Grown in a Greenhouse

Environmental Science and Pollution Control

Rawaa H.K. Al-Isawi, Miklas Scholz* and Furat A. M. Al-Faraj

Civil Engineering Research Group, School of Computing, Science and Engineering, The University of Salford, Newton Building, Salford M5 4WT, England, United Kingdom

***e-mail:m.scholz@salford.ac.uk; Tel.: 0044-161-2955921; fax: 0044-161-2955575

Online Resource 7 Overview of the outcome of the Chilli (C) harvest (before or on 24 December 2014) classification scheme

Inflow source	Class A	Class B	Class C	Class D	Class E	Mean Pence per plant
Filter 1	C1(217.3);C2(0.0); C3(181.2);C4(62.6); C5(111.2);C6(37.2)	C1(21.2);C2(4.8); C3(5.2);C4(32.5); C5(27.7);C6(15.8)	C1(14.9);C2(13.2); C3(28.9);C4(14.7); C5(26.3);C6(0.0)	C1(57.6);C2(12.9); C3(10.8);C4(17.5); C5(18.2);C6(12.4)	C1(0);C2(0); C3(0);C4(0); C5(0);C6(0)	157.4
Filter 2	C7(614.4);C8(536.9); C9(501.6);C10(507.7); C11(507.1);C12(97.5)	C7(155.2);C8(140.4); C9(148.0);C10(74.8); C11(62.2);C12(24.0)	C7(57.1);C8(57.5); C9(12.9);C10(22.7); C11(33.8);C12(25.9)	C7(9.2);C8(9.6); C9(2.9);C10(4.2); C11(3.3);C12(4.7)	C7(0);C8(0); C9(0);C10(0); C11(0);C12(0)	602.3
Filter 3	C13(152.2);C14(40.0); C15(127.1);C16(92.3); C17(29.7);C18(0.0)	C13(20.8);C14(9.4); C15(9.7);C16(30.4); C17(10.4);C18(3.9)	C13(44.5);C14(57.9); C15(49.6);C16(54.6); C17(45.9);C18(29.0)	C13(19.5);C14(17.9); C15(69.2);C16(16.9); C17(6.5);C18(31.1)	C13(0);C14(0); C15(0);C16(0); C17(0);C18(0)	161.4
Filter 4	C19(305.4);C20(359.6); C21(525.2);C22(319.7); C23(297.4);C24(271.6)	C19(104.5);C20(134.1); C21(59.8);C22(87.2); C23(56.7);C24(114.0)	C19(51.3);C20(40.2); C21(11.6);C22(62.4); C23(22.5);C24(17.2)	C19(7.3);C20(15.1); C21(15.7);C22(3.2); C23(2.2);C24(4.3)	C19(0);C20(0); C21(0);C22(0); C23(0);C24(0)	481.36
Filter 5	C25(316.1);C26(228.3); C27(791.5);C28(550.0); C29(265.2);C30(306.6)	C25(52.2);C26(75.4); C27(116.5);C28(77.5); C29(115.2);C30(57.9)	C25(122.4);C26(134.5); C27(77.9);C28(65.9); C29(42.9);C30(20.0)	C25(4.3);C26(1.0); C27(5.6);C28(0.0); C29(3.7);C30(20.5)	C25(0);C26(0); C27(0);C28(0); C29(0);C30(0)	575.2
Filter 6	C31(1071.9);C32(704.4); C33(1041.2);C34(946.9); C35(324.4);C36(504.6)	C31(140.1);C32(92.5); C33(108.5);C34(94.4); C35(151.0);C36(47.8)	C31(54.9);C32(26.6); C33(49.1);C34(61.3); C35(60.4);C36(14.7)	C31(3.3);C32(3.4); C33(8.2);C34(14.0); C35(0.0);C36(0.0)	C31(0);C32(0); C33(0);C34(0); C35(0);C36(0)	920.6

Online Resource 7 (cont.)

Filter 7	C37(1171.9);C38(883.9); C39(766.8);C40(1138.1); C41(1086.5);C42(418.6)	C37(98.4);C38(69.7); C39(160.5);C40(110.5); C41(51.9);C42(11.2)	C37(37.0);C38(14.4); C39(30.4);C40(19.7); C41(7.1);C42(4.4)	C37(13.9);C38(3.7); C39(1.0);C40(16.0); C41(3.3);C42(0.0)	C37(0);C38(0); C39(0);C40(0); C41(0);C42(0)	1019.8
Filter 8	C43(120.5);C44(453.9); C45(283.9);C46(303.7); C47(241.1);C48(110.4)	C43(108.0);C44(157.6); C45(160.3);C46(166.4); C47(92.2);C48(24.8)	C43(40.3);C44(112.3); C45(49.3);C46(72.1); C47(54.6);C48(23.9)	C43(38.0);C44(5.8); C45(0.0);C46(6.1); C47(0.0);C48(0.0)	C43(0);C44(0); C45(0);C46(0); C47(0);C48(0)	437.5
Control A	C49(66.9);C50(89.3); C51(112.6);C52(34.2); C53(31.8);C54(18.0)	C49(10.0);C50(19.7); C51(17.8);C52(12.7); C53(12.7);C54(18.7)	C49(31.0);C50(15.0); C51(54.9);C52(17.0); C53(36.0);C54(33.3)	C49(6.0);C50(14.1); C51(10.6);C52(19.6); C53(10.0);C54(2.3)	C49(0);C50(0); C51(0);C52(0); C53(0);C54(0)	115.7
Control B	C55(121.7);C56(234.3); C57(162.0);C58(166.0); C59(389.3);C60(60.0)	C55(67.2);C56(75.6); C57(86.3);C58(72.2); C59(43.8);C60(11.8)	C55(46.2);C56(31.1); C57(15.2);C58(42.3); C59(19.7);C60(40.8)	C55(22.2);C56(7.2); C57(5.5);C58(8.0); C59(11.0);C60(1.2)	C55(0);C56(0); C57(0);C58(0); C59(0);C60(0)	290.1
Deionised water	C61(184.6);C62(88.6); C63(0.0);C64(77.5); C65(169.4);C66(0.0)	C61(26.5);C62(64.2); C63(70.9);C64(57.4); C65(50.8);C66(78.3)	C61(36.1);C62(40.2); C63(29.0);C64(14.4); C65(11.9);C66(21.4)	C61(9.3);C62(7.9); C63(6.9);C64(4.7); C65(12.0);C66(6.3)	C61(0);C62(0); C63(0);C64(0); C65(0);C66(0)	178.1
Tap water	C67(579.7);C68(562.2); C69(474.1);C70(624.3); C71(393.2);C72(503.3)	C67(115.4);C68(41.3); C69(37.4);C70(31.2); C71(62.1);C72(56.4)	C67(42.8);C68(46.3); C69(29.7);C70(30.5); C71(31.9);C72(30.7)	C67(3.5);C68(0.0); C69(0.0);C70(1.0); C71(1.2);C72(1.6)	C67(0);C68(0); C69(0);C70(0); C71(0);C72(0)	616.6
Tap water/fertiliser	C73(867.3);C74(1340.4); C75(540.3);C76(850.1); C77(561.2);C78(977.9)	C73(80.0);C74(118.4); C75(91.8);C76(85.4); C77(74.1);C78(138.7)	C73(28.5);C74(62.0); C75(61.7);C76(22.8); C77(7.4);C78(56.0)	C73(2.0);C74(3.4); C75(3.0);C76(0.9); C77(10.1);C78(2.1)	C73(0);C74(0); C75(0);C76(0); C77(0);C78(0)	997.6
Wastewater/tap	C79(1050.8);C80(632.9); C81(1504.1);C82(1238.8); C83(812.0);C84(1420.6)	C79(108.7);C80(79.7); C81(163.9);C82(101.6); C83(122.7);C84(72.1)	C79(54.5);C80(40.0); C81(33.2);C82(10.1); C83(35.4);C84(42.0)	C79(0.0);C80(4.5); C81(1.2);C82(0.0); C83(4.2);C84(5.0)	C79(0);C80(0); C81(0);C82(0); C83(0);C84(0)	1256.3
Wastewater	C85(345.5);C86(421.7); C87(603.2);C88(424.4); C89(657.8);C90(698.2)	C85(121.2);C86(146.6); C87(106.8);C88(93.3); C89(127.9);C90(96.6)	C85(115.6);C86(58.6); C87(48.3);C88(15.3); C89(46.7);C90(70.8)	C85(8.7);C86(19.2); C87(1.7);C88(0.0); C89(1.2);C90(11.8)	C85(0);C86(0); C87(0);C88(0); C89(0);C90(0)	706.9

Note that the lowest variable class entry for any individual fruit assessment will determine the final class. However, only the following numerical and objective variables were used to classify fruits for the purpose of this study: length, width, weight and bending. Values shown per plant represent pence (Sterling)
